# Author Correction: Porous organic polycarbene nanotrap for efficient and selective gold stripping from electronic waste

**DOI:** 10.1038/s41467-023-36417-z

**Published:** 2023-02-06

**Authors:** Xinghao Li, Yong-Lei Wang, Jin Wen, Linlin Zheng, Cheng Qian, Zhonghua Cheng, Hongyu Zuo, Mingqing Yu, Jiayin Yuan, Rong Li, Weiyi Zhang, Yaozu Liao

**Affiliations:** 1 State Key Laboratory for Modification of Chemical Fibers and Polymer Materials, College of Materials Science and Engineering, Donghua University, Shanghai, 201620 China; 2 Department of Materials and Environmental Chemistry, Stockholm University, Stockholm, 10691 Sweden; 3 College of Chemistry and Chemical Engineering, Donghua University, Shanghai, 201620 China

**Keywords:** Pollution remediation, Pollution remediation, Chemical engineering

Correction to: *Nature Communications* 10.1038/s41467-023-35971-w, published online 17 January 2023

The original version of this Article contained a typographical error in the affiliation details for Authors Xinghao Li, Jin Wen, Linlin Zheng, Cheng Qian, Zhonghua Cheng, Hongyu Zuo, Mingqing Yu, Weiyi Zhang, and Yaozu Liao, which was previously incorrectly given as ‘1State Key Laboratory for Modification of Chemical Fibers and Polymer Materials, College of Materials Science and Engineering, Donghua University, Shanghai, 201620, China’ but should have been ‘State Key Laboratory for Modification of Chemical Fibers and Polymer Materials, College of Materials Science and Engineering, Donghua University, Shanghai, 201620, China’.

Furthermore, in the original Methods section, one of the subheadings also contained a typographical error and was incorrectly given as ‘Synthesis of 4-cyanomethy-1-viny-1,2,4-triazolium bromide’ but should have been ‘Synthesis of 4-cyanomethyl-1-vinyl-1,2,4-triazolium bromide’.

These have been corrected in both the PDF and HTML versions of the Article.

