# Supplementary material for: Porous organic polycarbene nanotrap for efficient and selective gold stripping from electronic waste
Source: Nat Commun. 2023 Jan 17;14:263. doi: 10.1038/s41467-023-35971-w (PMC9845340; doi:10.1038/s41467-023-35971-w)
Supplement: Supplementary file 1 — Supplementary Information [file 41467_2023_35971_MOESM1_ESM.pdf]

Supplementary Information

Porous Organic Polycarbene Nanotrap for Efficient and Selective Gold Stripping from Electronic Waste

Xinghao Li[1], Yong-Lei Wang[2], Jin Wen[1], Linlin Zheng[1], Cheng Qian[1], Zhonghua Cheng[1], Hongyu Zuo[1], Mingqing Yu[1], Jiayin Yuan[2], Rong Li[3], Weiyi Zhang[1]* & Yaozu Liao[1]*

[1]State Key Laboratory for Modification of Chemical Fibers and Polymer Materials, College of Materials Science and Engineering, Donghua University, Shanghai 201620, China

[2]Department of Materials and Environmental Chemistry, Stockholm University, Stockholm 10691, Sweden

[3]College of Chemistry and Chemical Engineering, Donghua University, Shanghai 201620, China

*Corresponding authors: Weiyi Zhang (wyzhang@dhu.edu.cn)

Yaozu Liao (yzliao@dhu.edu.cn)

Supplementary Methods

1. Characterization methods

The liquid proton nuclear magnetic resonance (^1H NMR) measurements and ^{13}C nuclear magnetic resonance (^{13}C NMR) measurements were taken at a Bruker Advance 600 (600 MHz) spectrometer. The solid-state ^{13}C nuclear magnetic resonance (^{13}C NMR) measurements were taken at a Bruker Advance 400 (400 MHz) spectrometer. Fourier-transform infrared spectroscopy (FT-IR) spectra were collected using a Thermo Scientific Nicolet iS5 spectrometer. The scanning electron microscope (SEM) images were obtained on a Hitachi SU8010 microscope. The transmission electron microscope (TEM) images were obtained on a Talos F200S microscope. Aberration-correction high-angel annular dark field scanning transmission electron microscopy (AC-HAADF-STEM) images and the energy dispersive spectroscopy (EDS) were recorded on a FEI Tecnai Osiris and Titan3. The specific surface areas of the samples were determined according to the Brunauer-Emmett-Teller (BET) model and the pore size distribution was calculated by the Non-Local Density Functional Theory (NLDFT) method. All relative data were obtained on a Micro ASAP 2046 surface area and porosity analyzer. X-ray photoelectron spectroscopy (XPS) was conducted at a Thermo Fisher Scientific Escalab 250Xi under high vacuum ($1*10^{-9}$ Torr), and all binding energy were calibrated to the C1s peak at 284.8eV. The crystallinity of the samples that after adsorbed metal ions was characterization by an Arigaku D Max 2550 VB PC X-ray diffraction spectrometer (18 KW, Cu Kα radiation with λ=1.548 Å). The concentration of Au^{3+} and other metal elements was measured on NexlON 350D inductively coupled plasma mass spectrometer (ICP-OES PerkinElmer). The number average molecular weight (Mn) and polydispersity (PDI) of samples were measured on Breeze gas gel

chromatograph (Waters, USA). The water contact angle of materials were measured by OCA25 video optical contact angle meter (Dataphysics, Germany).

2. Gold adsorption and recovery study

2.1 Adsorption isothermal experiments

The Langmuir adsorption isotherm model and the Freundlich adsorption isotherm model was applied to study the adsorption behavior of metal ions on adsorbent. As an empirical model, Freundlich adsorption model is based on the assumption that adsorption sites with strong adsorption capacity preferentially complete the adsorption process, resulting in the formation of multilayers on heterogeneous surfaces and the interaction between adsorbed ions. The adsorption isotherm model can be described mathematically as Equation (1).

$$Qe=k_F\ C_e^{1/n} \quad (1)$$

Where C_e is the equilibrium concentration (mg/L), Qe is the equilibrium adsorption capacity (mg/g), *n* is the Freundlich exponent, k_F (mg/g) is the Freundlich adsorption isotherm constant.

The Langmuir adsorption model assumes that all adsorption sites in the adsorbent are equivalent, which forms a homogeneous surface with the same force on the surface of the adsorbent. The adsorption isotherm model can be described mathematically as Equation (2).

$$\frac{C_e}{Q_e}=\frac{1}{Q_{max}\times k_L}+\frac{C_e}{Q_{max}} \quad (2)$$

Where Ce is the equilibrium concentration (mg g^{-1}), Qe is the equilibrium adsorption capacity (mg g^{-1}), Q_{max} is the maximum adsorption amount at the time of equilibrium (mg g^{-1}), k_L (L mg^{-1}) is the Langmuir adsorption isotherm constant.

To obtain adsorption isotherms, 5 mg of Ptriaz-CN-A was placed in 10 mL aqueous solutions of varying Au^{3+}/Pt^{2+} concentrations (100-5000 ppm). The solutions were then stirred for 24 h at 600 rpm to achieve adsorption equilibrium. The solutions were filtered through a 0.45 μm syringe filter units and the filter was analyzed *via* ICP-OES to determine the residual metal ion concentrations. The amount adsorbed or uptake capacity, Qe (mg g^{-1}), at equilibrium was calculated using Equation (3).

$$Q_e = \frac{(C_0 - C_e) \times V}{m} \quad (3)$$

Where C_0 (mg L^{-1}) and C_e (mg L^{-1}) are the original concentration and adsorption equilibrium concentration, respectively. V is the volume of solution (L), m is the mass of adsorbent (g).

2.2 pH effect on gold adsorption

100 ppm Au^{3+} solution was adjusted by KOH (1 M) and HCl (1 M) to pH values of 2, 4, 7, 9. At each pH, six experiments samples were prepared. Each sample was added 10 mL of the solution and 5 mg of Ptriaz-CN-A was added to each experiment sample. The tubes were then shaken at 300 rpm for 30 min, 1 h, 3 h, 6 h, 12 h, and 24 h. The adsorbent was then filtered by 0.45 μm syringe filter units and the remaining Au^{3+} concentrations in solution were determined by ICP-OES.

2.3 Desorption and recyclability

100 ppm Au^{3+} solution was used for adsorption steps. The adsorption experiments were performed as described in the Au^{3+} adsorption isotherm experiment above. After one run of adsorption, the Ptriaz-CN-A (Au) was regenerated by treatment with the elution solution and reused for another adsorption experiment. In a typical desorption experiment, the Ptriaz-CN-A (Au) was first rinsed with DI water, and then desorbed with 20 mL elution solution containing a 1:1 (v/v) solution of thiourea (1 M) and HCl (1 M) under stirring for at 60 °C for 8 h. The filtrate was separated from

the adsorbent by filtration and the concentration was determined by ICP-OES. The elution efficiency was determined by using Equation (4)

$$\text{Elution Efficiency}=\frac{C_e\times V_e}{(C_o\text{-}C_t)\times V_a}\times 100\ \% \qquad (4)$$

Where C_e (mg L^{-1}) is the Au^{3+} concentration in the elution solution, Ve (L) is the volume of elution solution, C_t (mg L^{-1}) is the Au^{3+}concentration in water after Au^{3+} adsorption, C_o (mg L^{-1}) is the original Au^{3+} concentration in water, Va (L) is the volume of Au^{3+} contained water for adsorption.

2.4 Selectivity in multi-element solutions

5 mg of Ptriaz-CN-A was added to tube containing 10 mL of a mixed metal solution containing 5 ppm Au^{3+}, Pt^{2+}, and 300 ppm Cu^{2+}, Mg^{2+}, Ni^{2+}, Cr^{2+}, Co^{2+}, and Zn^{2+}. The solutions were stirred overnight to achieve equilibrium. And then filtered through a 0.45 μm membrane filter, the filtrate was analyzed *via* ICP-OES to determine the residual metal ion concentrations. Comparison of the selectivity adsorption experiments were performed using the following Equation (5) and Equation (6)

$$\text{Recovery Efficiency}=\frac{C_o-C_t}{C_o}\times 100\ \% \qquad (5)$$

Where C_0 (mg L^{-1}) and C_t (mg L^{-1}) are the original concentration and the concentration at a given time of metal ions.

$$K_d=\frac{(C_0\text{-}C_e)}{C_e}\times\frac{V}{m} \qquad (6)$$

Where K_d is the distribution coefficient describing the affinity of the adsorbent toward a certain metal ion; C_0 (mg L^{-1}) and C_e (mg L^{-1}) are the original concentration and adsorption equilibrium concentration, and V is the volume of solution (L), m is the mass of adsorbent (g); the selectivity coefficient of two metal ions, M1 / M2, is represented by the ratio of K_d values of the according metal ions.

2.5 Gold capture from actual e-waste solution

We choose disused CPU as waste electrical and electronic equipment. The

waste CPU which we used had an array of pins composed of Au, Cu and some other metals. Then, the metal spins were removed from CPU to be processed in leaching solution. The metal leaching solution was prepared by mixing 1 μL of pyridine and 750 mg of NBS in 120 mL of DI water. 0.4 g of the metal spins were put into the solution and the mixture was let stand for 4 days. The resulting e-waste solution was then filtrated and acidified by 1 M HCl to pH=2. The metal content and concentration of the solution was determined by ICP-OES. The adsorption experiment with Ptriaz-CN-A was then performed following the procedure described in the metal selectivity test mentioned above.

2.6 Extraction of trace amounts of gold from water

The adsorbents' ability to concentrate trace amounts of gold over time was also tested. For this, 5 mg of Ptriaz-CN-A was added to a 50 mL solution containing 100 ppb Au^{3+}. The solutions were then stirred for 24 h and 48 h at 600 rpm, respectively. After that, the solutions were filtered through a 0.45 μm syringe filter units and the filter was analyzed *via* ICP-OES to determine the residual Au^{3+} concentrations.

3. Theoretical calculations

We have employed the Gaussian 09 Revision D.01[1] and Multiwfn (version 3.8) [2, 3]to perform all density functional theory (DFT) calculations at TPSSH/def2-SVP and TPSSH/def2-TZVP levels to elucidate the adsorption-reduction mechanism of Ptriaz-CN-A[4, 5].

3.1 Energy calculations

A single ammonia crosslinking Ptriaz-CN-A unit was used as the model structure to calculate the adsorption and binding energies of metal ions on Ptriaz-CN-A on the basis of Equation (7)

$$E_{bind/ads}=E_{configuration}-E_{Ptriaz\text{-}CN\text{-}A}-E_{Metal} \quad (7)$$

Where $E_{configuration}$, $E_{Ptriaz\text{-}CN\text{-}A}$, and E_{Metal} represent the energy of the Ptriaz-CN-A adsorbed with metal ions, isolated ammonia crosslinking Ptriaz-CN-A model unit and metal ions in the optimized geometries, respectively. It should be noted that $E_{configuration}$, $E_{Ptriaz\text{-}CN\text{-}A}$, E_{Metal} should be calculated at the same basis set level, otherwise, BSSE should be took into consideration.

In general, the weak interaction energy between molecules A and B cannot be calculated simply through $E_{interaction} = E_{AB} - E_A - E_B$, because the decrease of E_{AB} energy relative to $E_A + E_B$ comes from two aspects. On the one hand, it is the real interaction energy between molecules A and B. That's what we want; On the other hand, the basic functions of molecules A and B overlap in the complex system, which is equivalent to increasing the basis group of the complex and reducing the energy of E_{AB} (strictly speaking, the premise is that the theoretical method used is based on the variational principle). If this part of contribution is also incorporated into $E_{interaction}$, the interaction energy is overestimated (that is, the binding energy is actually not as negative as calculated), so it should be removed, which is called the Basis Set Superposition Error (BSSE). Therefore, the interaction energy of two molecules should be formulated as $E_{interaction} = E_{AB} - E_A - E_B + E_{BSSE}$. For weak interactions, the proportion of E_{BSSE} in $E_{interaction}$ is often not small, or even exceeds it. If not corrected, the positive and negative signs may not be correct.

For the process of Au^{3+} adsorption-reduction on model unit of Ptriaz-CN-A, the energy difference (ΔE) in the adsorption process was defined as $E_{products}$-$E_{reactants}$, on the basis of Equation (8) to Equation (11), where E is the electronic energy.

$$\Delta E_1=[E_{Ptriaz\text{-}CN\text{-}A\,(AuCl_4^-)}+2E_{H_3O^+}]-[E_{Ptriaz\text{-}CN\text{-}A}+E_{AuCl_4^-}+2E_{H_3O^+}] \quad (8)$$

$$\Delta E_2=[E_{Ptriaz\text{-}CN\text{-}A\,(AuCl_2^-)}+2E_{H_3O^+}+2E_{HCl}]-[E_{Ptriaz\text{-}CN\text{-}A\,(AuCl_4^-)}+2E_{H_3O^+}] \quad (9)$$

$$\Delta E_3=[E_{Ptriaz\text{-}CN\text{-}A\,(Au)}+2E_{H_2O}+2E_{HCl}]-[E_{Ptriaz\text{-}CN\text{-}A\,(AuCl_2^-)}+2E_{H_3O^+}] \quad (10)$$

$$\Delta E_4=[E_{Ptriaz\text{-}CN\text{-}A\,(Au\text{-}AuCl_4^-)}+2E_{H_2O}]-[E_{Ptriaz\text{-}CN\text{-}A\,(Au)}+2E_{H_2O}+E_{AuCl_4^-}] \quad (11)$$

All the structures are geometrically optimized by TPSSH/def2-SVP, and used TPSSH/def2-TZVP to calculate the energy.

3.2 Chemical values calculations

When the spin multiplicity is 1, according to the Koopmans theory, hardness = fundamental gap ≈HOMO-LUMO gap, VIP ≈ -E(HOMO), VEA ≈ -E(LUMO), Mulliken electronegativity ≈ -1/2 (VIP+VEA), chemical potential ≈ 1/2 (VIP+VEA).

When the spin multiplicity is 2, conceptual density functional theory (CDFT), we can directly calculate the wave function and energy of N, N+1, N-1 electronic states, and then obtain the parameters.

4. Streamlined life cycle assessment (LCA)

Environmental impacts of Ptriaz-CN-A production were analyzed using the "cradle-to-gate" LCA approach. LCA is a systematic tool for determining the environmental impact of a product or a process across its entire life cycle or a portion of its life cycle (ISO 14040, ISO 14044). The life cycle environmental impacts were modeled using Simapro software (version 9.0) with Ecoinvent databases as inventories. TRACI 2.1 (tool for the reduction and assessment of chemical and other environmental impacts) was used as the assessment method. For this study, ten impact categories have been selected: (a) ozone depletion (OD; kg CFC-11 equivalent), (b) global warming potential (GW; kg CO_2 eq), (c) smog (SM; kg O_3 eq), (d) acidification (AC; mol SO_2 eq), (e) eutrophication (ET; kg N eq), (f) carcinogenic (HHC; CTUh), (g) noncarcinogenic (HHNC; CTUh), (h) respiratory effects (RE; kg PM2.5 eq), (i) ecotoxicity (EC; CTUe), and (j) fossil fuel depletion (FFD; MJ surplus). It should be noted that some parameters are not considered due to the lack of source data of relevant

chemicals/processes. Therefore, it should be treated with caution when applying for other synthetic processes or materials.

4.1 System boundary and functional unit

Overall system boundary used in the current study is depicted in Fig. S38. The stage consists of six major stages: (1) synthesis of TriazoleBr, (2) synthesis of PtriazBr, (3) synthesis of Ptriaz-CN, (4) TIPS process, (5) crosslink & solvent exchange process and (6) drying process. Considering the actual production conditions of the laboratory, we collect LCA data based on the purpose of laboratory research Scale-1: small dose feeding, supercritical CO_2 drying as drying method; Scale-2: data based on the maximum production scale of the laboratory. In Scale-2, considering that the supercritical CO_2 drying equipment in the laboratory can only handle limited amounts of samples each time, freeze-drying is chosen as the final drying method. As presented in the Fig. S37, the inputs (raw materials and electricity usage) and outputs (1 g Ptriaz-CN-A) have been listed. Furthermore, 1 g Ptriaz-CN-A production was set as the functional unit.

4.2 Data collection and inventory

The Life Cycle Inventory (LCI) was obtained by collecting inventory data from the laboratory logs, analytical procedures, and material energy balances. The uncertainty analysis was performed using Monte Carlo simulation with uniform distribution of the range of parameters with 1000 iterations. Furthermore, uncertainty range from the Monte Carlo simulation, where the upper bound representing the 95 percentile and the lower bound representing the 5 percentile are also include. The detailed information about LCI is presented in Table S11 to Table S15.

4.3 Discussions

Environmental impacts of Ptriaz-CN-A production prepared at different scales were assessed. The relative results are provided in the main text, Fig.

6a. It is clear that the Ptriaz-CN-A prepared at Scale-2 has much lower environmental impacts across all categories. This is due to the reason that equipment can be operated under full load conditions and resources can be maximized. This indicates that there is much room to reduce the environmental impact of Ptriaz-CN-A production when they are produced on an industrial scale.

As shown in Fig. S38a and Fig. S38b, for ozone depletion, the proportion of environmental influence of Synthesis Stage-3 phase is relatively large, accounting for 58 % and 94 % in Scale-1 and Scale-2, respectively (Table S16 and Table S23). Moreover, in the OD impact category of Synthesis Stage-3, LiTFSI has the highest proportion of environmental impact, contributes more than 99 % (Table S19 and Table S26). This may be attributed to a large amount of LiTFSI is used in this process to exchange counterions, and TFSI anion containing F in aqueous solution may be discharged into the environment as ozone depleting substances. Of the other nine impact categories besides OD, the Synthesis Stage-1 and Drying process phases have a greater proportion of environmental impact, contribute 29 % ~ 42 % and 31 % ~ 43 % in Scale-1, 24 % ~ 43 % and 15 %~34 % in Scale-2, respectively (Table S17, Table S22, Table S24 and Table S29). This can be attributed to the use of 1-vinyl-1,2,4-traizole and bromoacetonitrile, which may be harmful to the environment. Moreover, the relatively higher power consumption of equipment during synthesis and drying process can also have an impact on the environment. For Synthesis Stage-2, TIPS Process and Crosslink & Solvent exchange Process, the environmental impacts of these preparation process in the Ptriaz-CN-A production are relatively small, contribute approximately 1 %~16 %, 2 %~22 % and 0.2 %~30 % environmental impact proportions among the other nine impact categories (Table S16 and Table S23).

5. Cost analysis

Cost analysis of raw materials and electricity in Ptriaz-CN-A preparation process in two different scales are presented in the main text, Fig. 6b, the detailed data are summarized in Table S30 and Table S31, along with the corresponding calculated production energy. All data (price of electricity in Shanghai, price of raw materials and gold price in China) were obtained in October, 2022. And all data were at the lab-scale. From Fig. 6b, the production cost for synthesis of 1 g Ptriaz-CN-A in Scale-1 and Scale-2 is around 116.988 CNY and 107.280 CNY, respectively, and more than 95 % of which are spent on raw materials. Although this production cost seems high, it should be noted that the cost here is based on our laboratory data. It is clear that the cost preparation is reduced when we scale up production under laboratory conditions (Scale-2). Therefore, the preparation cost of this adsorbent can be greatly reduced when it is mass-produced in the industry. For instance, reducing the cost of 1-vinyl-1,2,4-triazole preparation from the source in industry is crucial to reduce the production cost of Ptriaz-CN-A. Now, commercially available 1-vinyl-1,2,4-triazole is mainly obtained by the addition reaction of acetylene to triazoles. Though, the required synthetic source materials, 1,2,4-triazole and acetylene, are particularly cheap, it is possible that 1,2,4-triazole is not yet widely used, and the small batch production of this monomer in the Lab make this monomer very expensive (Table. S32). Instead, the 1-vinyl imidazole prepared in the same way is so widely used in industry that large-scale preparation makes it very cheap. Therefore, on the basis of the above-mentioned considerations, Ptriaz-CN-A would have broad prospects of application to new adsorbing agent.

Supplementary Figures

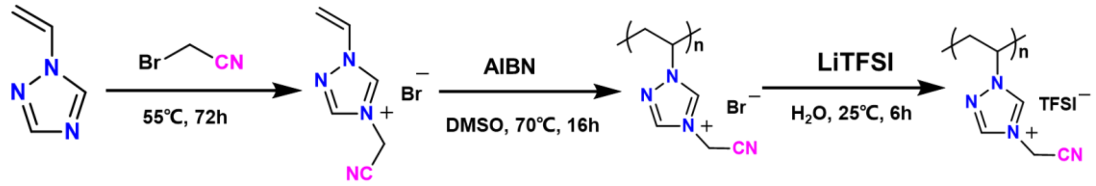

Fig. S1 Schematic illustration of synthesis procedures for Ptriaz-CN

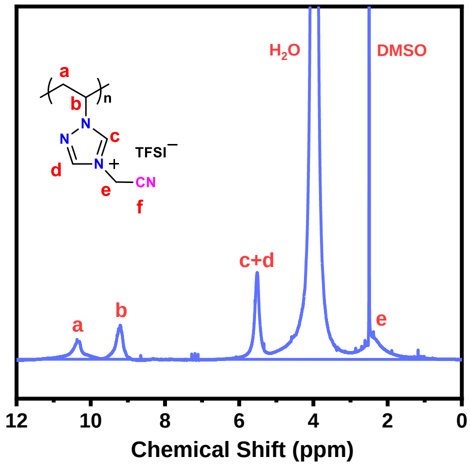

Fig. S2 ^{1}H NMR spectra of poly(4-cyanomethyl-1-vinyl-1,2,4-triazolium bis(trifluoromethanesulfonyl)imide) (Ptriaz-CN). Source data are provided as a Source Data file.

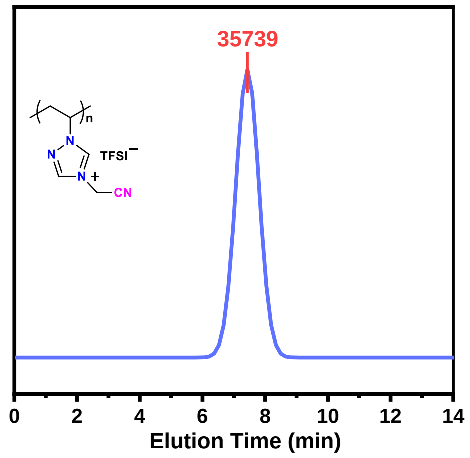

Fig. S3 GPC curves measured (in DMF) for Ptriaz-CN. (The apparent number-average molecular weight and PDI value are 35739 g/mol and 2.14, respectively.) Source data are provided as a Source Data file.

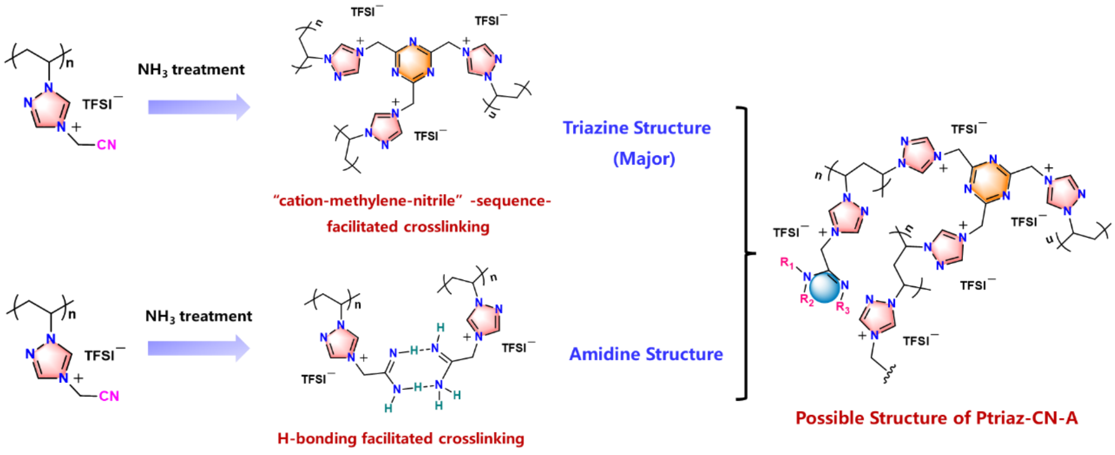

Fig. S4 Chemical reactions with both amidine and triazine structures for Ptriaz-CN-A formation paths.

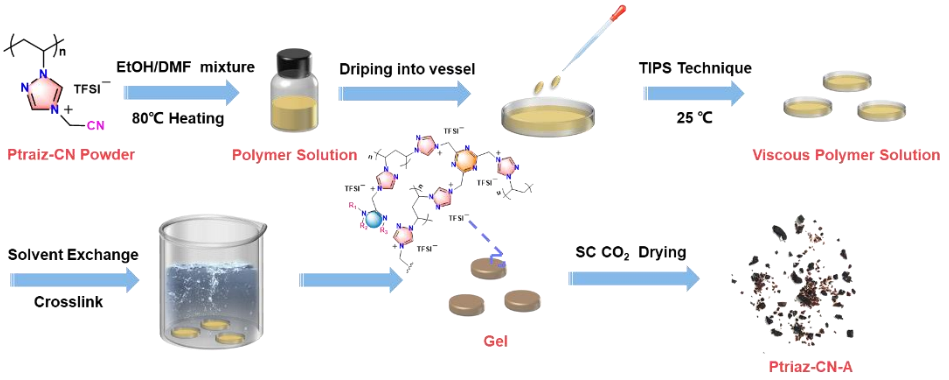

Fig. S5 General pathway toward mesoporous Ptriaz-CN-A

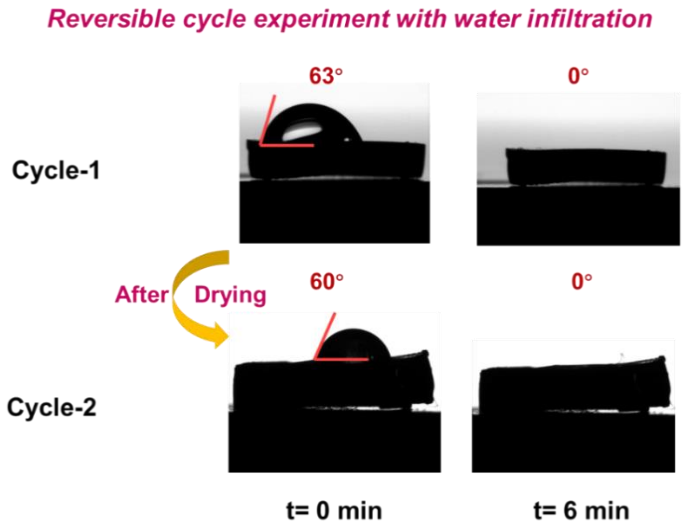

Fig. S6 Wetting process and water contact angle measurement of the Ptriaz-CN-A. It took 6 min for the contact layer to change from the hydrophobic to the hydrophilic.

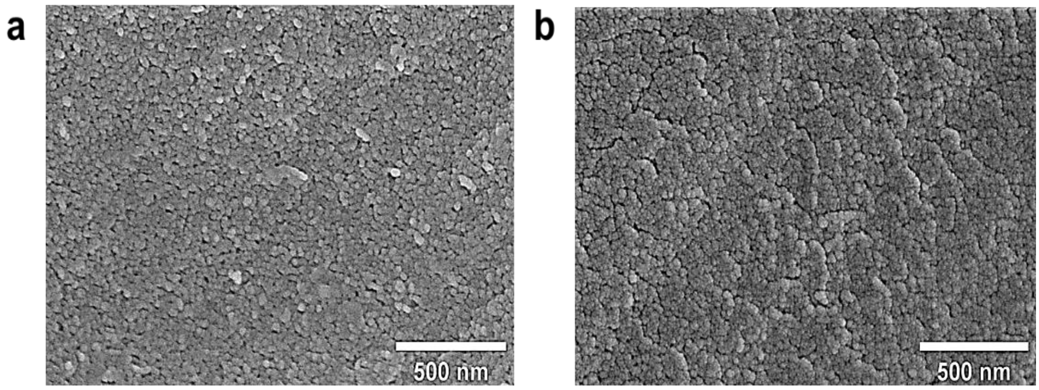

Fig. S7 The SEM images of Ptriaz-CN-A. (a) Original Ptriaz-CN adsorbent, (b) Ptriaz-CN adsorbent after water infiltration.

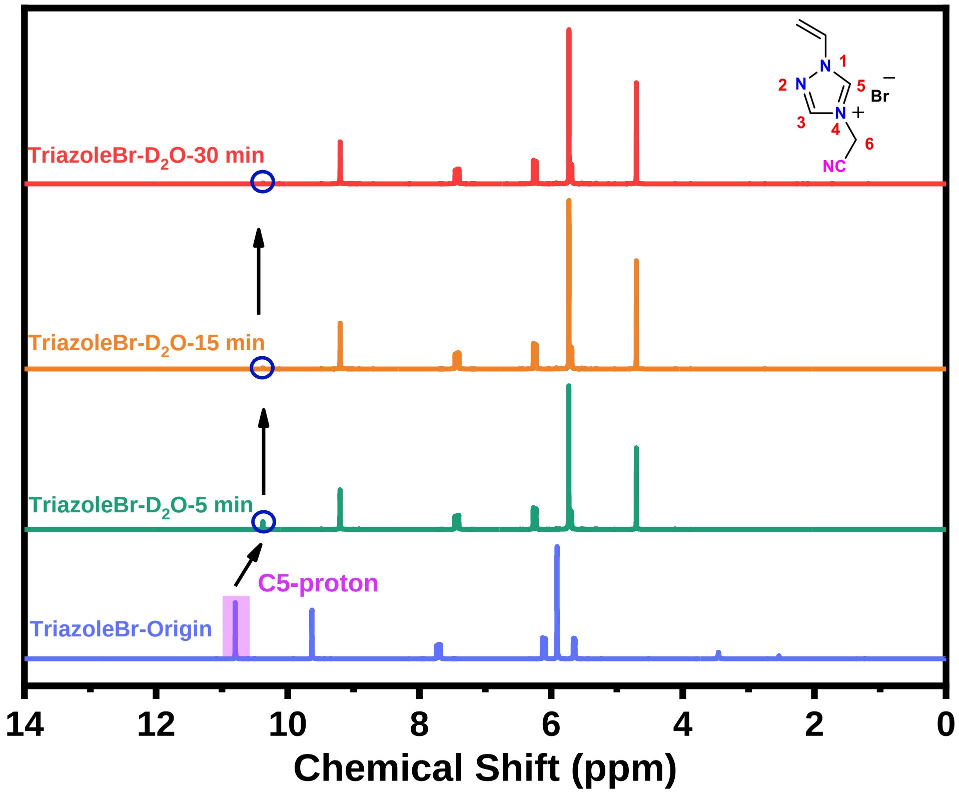

Fig. S8 Time-dependent ^{1}H-NMR test of proton exchange of TriazoleBr. Source data are provided as a Source Data file.

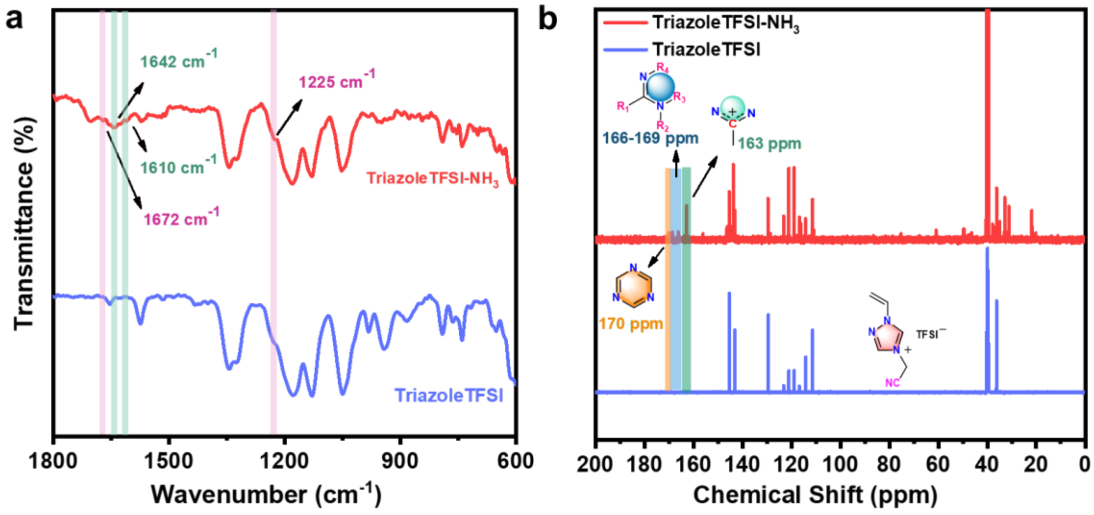

Fig. S9 (a) FT-IR and **(b)** ^{13}C NMR spectra of unpolymerized precursor-TriazoleTFSI (bottom line) and NH_3 treated TriazoleTFSI (top line). Source data are provided as a Source Data file.

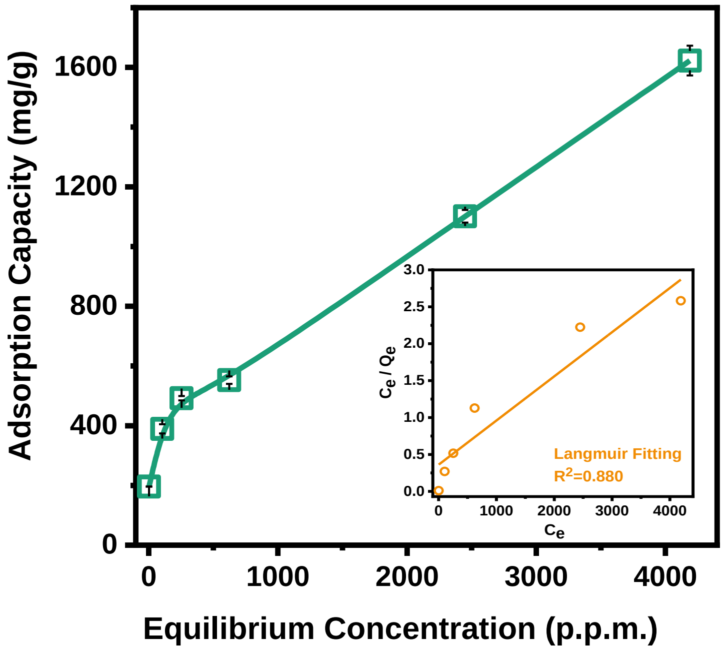

Fig. S10. Adsorption isotherm of Pt^{2+} on Ptriaz-CN-A and related Langmuir isotherm fitting (R^2=0.880). Error bars represent standard deviation. n=3 independent experiments. Source data are provided as a Source Data file.

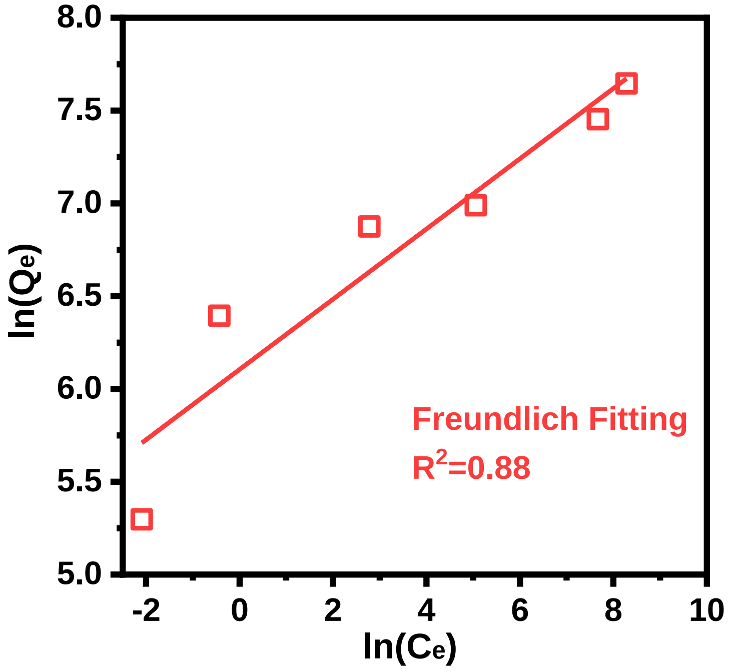

Fig. S11 Freundlich adsorption isotherm fitting of Au^{3+} on Ptriaz-CN-A. Source data are provided as a Source Data file.

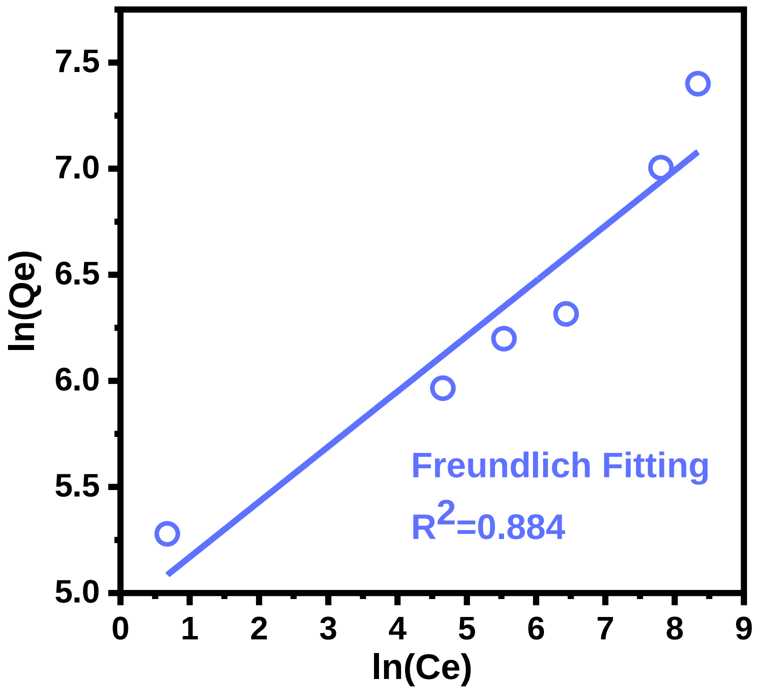

Fig. S12 Freundlich adsorption isotherm fitting of Pt^{2+} on Ptriaz-CN-A. Source data are provided as a Source Data file.

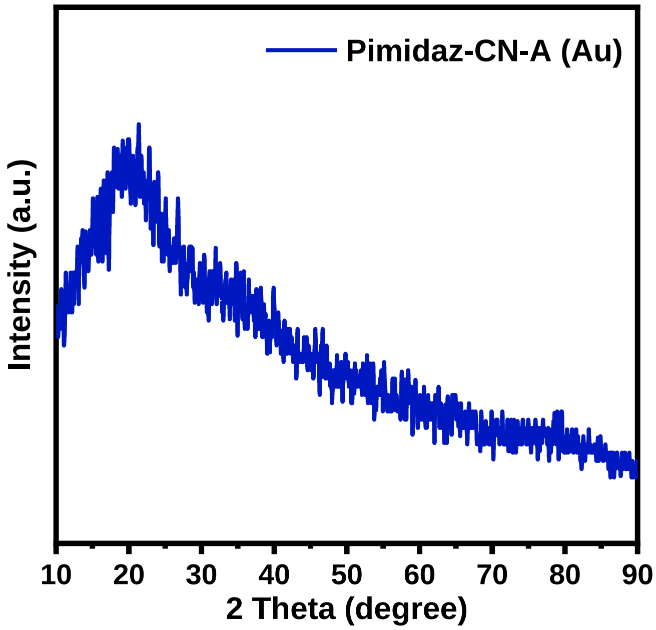

Fig. S13 XRD patterns for Au-loaded Pimidaz-CN-A. Source data are provided as a Source Data file.

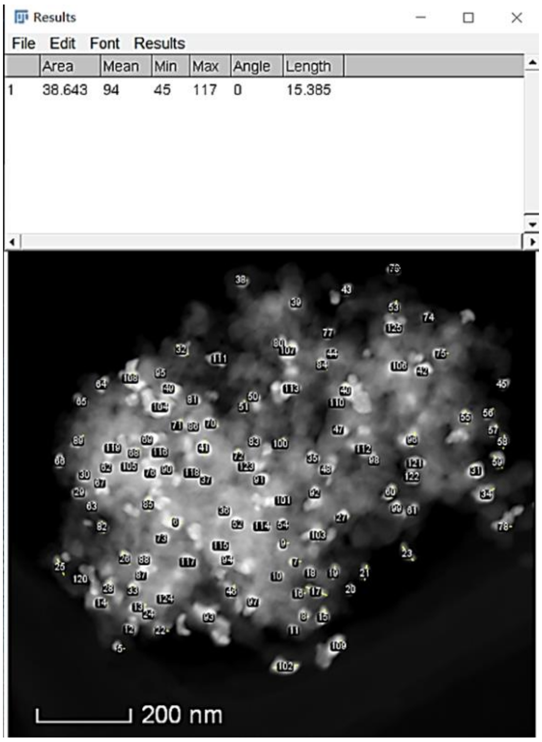

Fig. S14 Statistics on the Au particle size of Au-loaded Ptriaz-CN-A by ImageJ software.

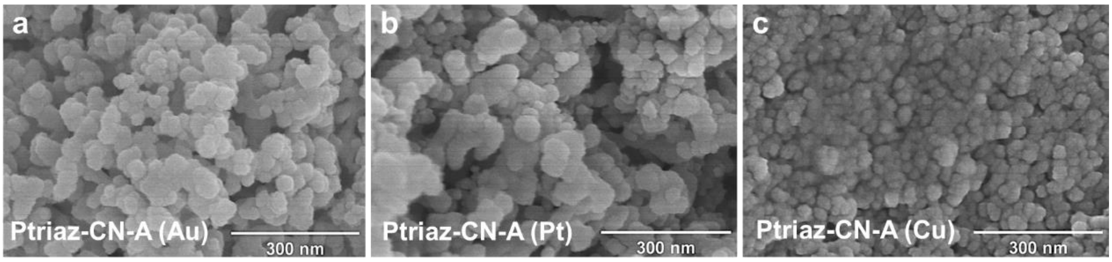

Fig. S15 The SEM images of metal loaded Ptriaz-CN-A. **(a)** Ptriaz-CN-A (Au), **(b)** Ptriaz-CN-A (Pt), **(c)** Ptriaz-CN-A (Cu).

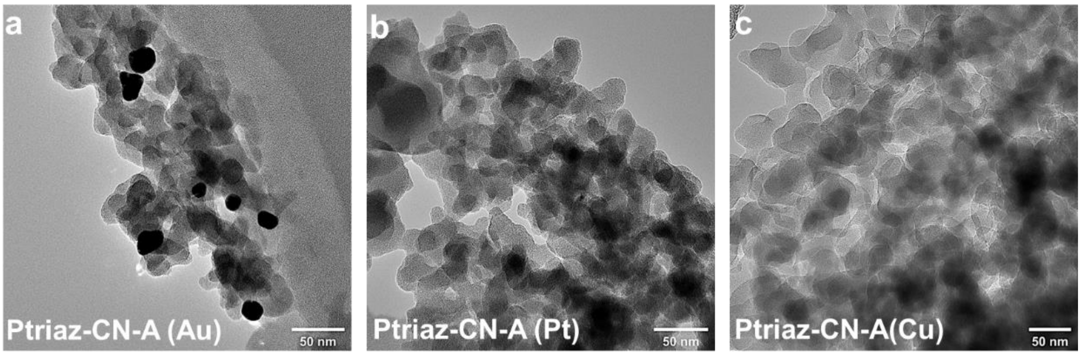

Fig. S16 The TEM images of metal loaded Ptriaz-CN-A. **(a)** Ptriaz-CN-A (Au), **(b)** Ptriaz-CN-A (Pt), **(c)** Ptriaz-CN-A (Cu).

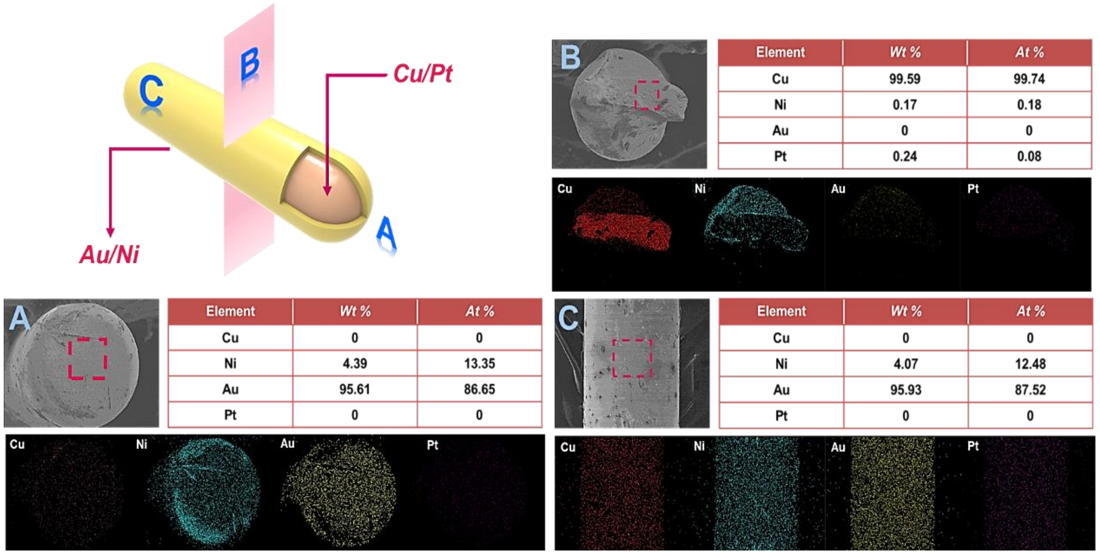

Fig. S17 The corresponding EDX and SEM mapping spectra showing the elemental composition on the point of pin (A), cross section of pin (B) and surface of pin (C).

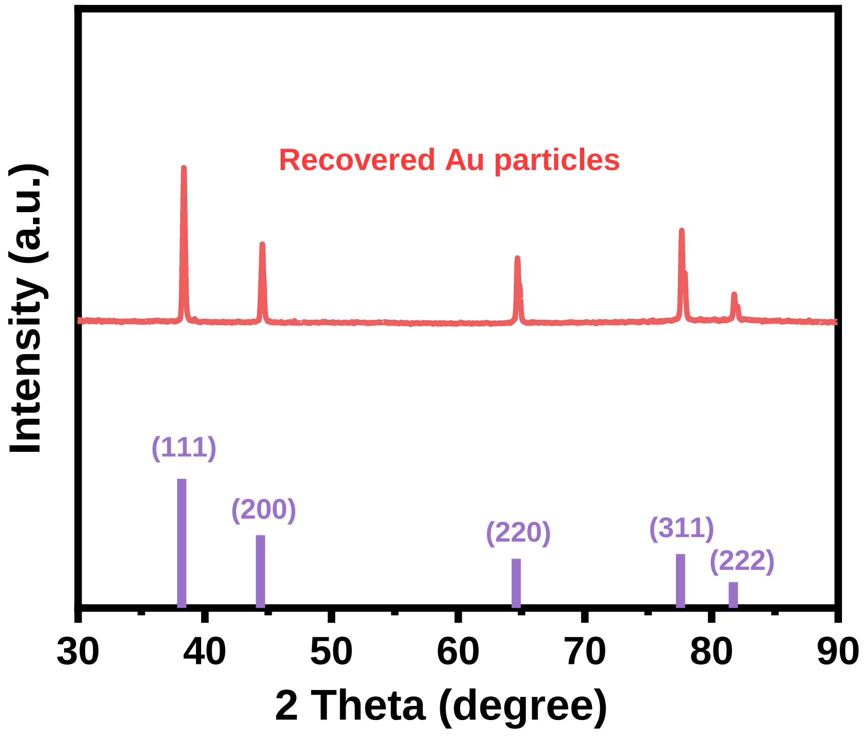

Fig. S18 XRD pattern of Au nanoparticles that was purified from Ptriaz-CN-A. The pattern indicates that Au^{3+} is reduced to Au^{0} after treatment with the Ptriaz-CN-A. Source data are provided as a Source Data file.

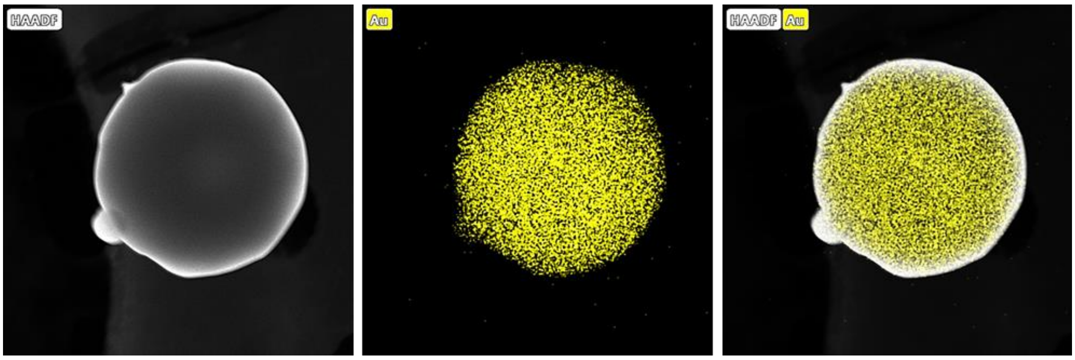

Fig. S19 STEM image and elemental mapping for recovered gold particles.

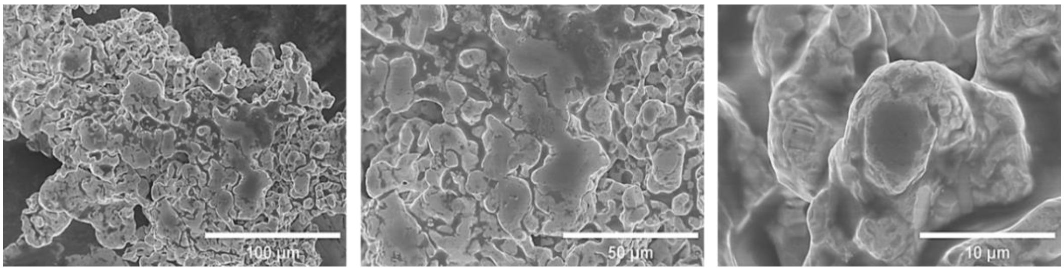

Fig. S20 SEM images of recovered gold particles.

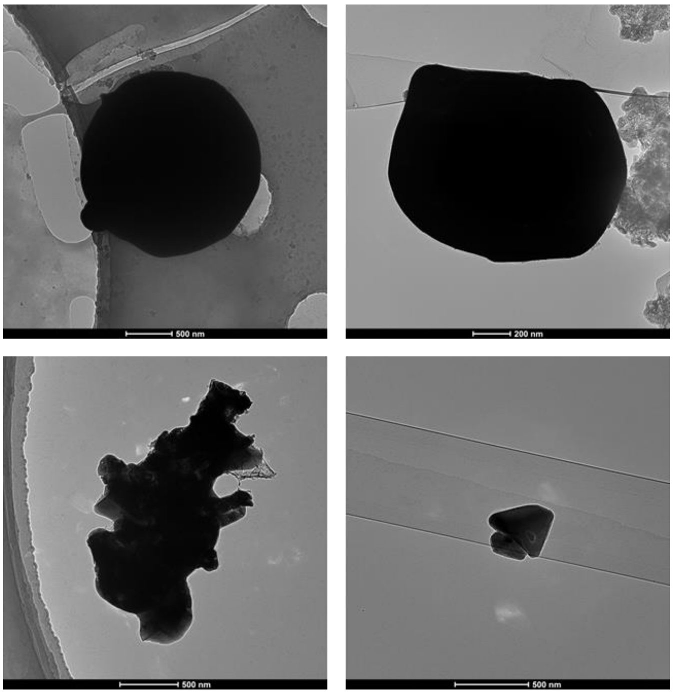

Fig. S21 TEM images of recovered gold particles.

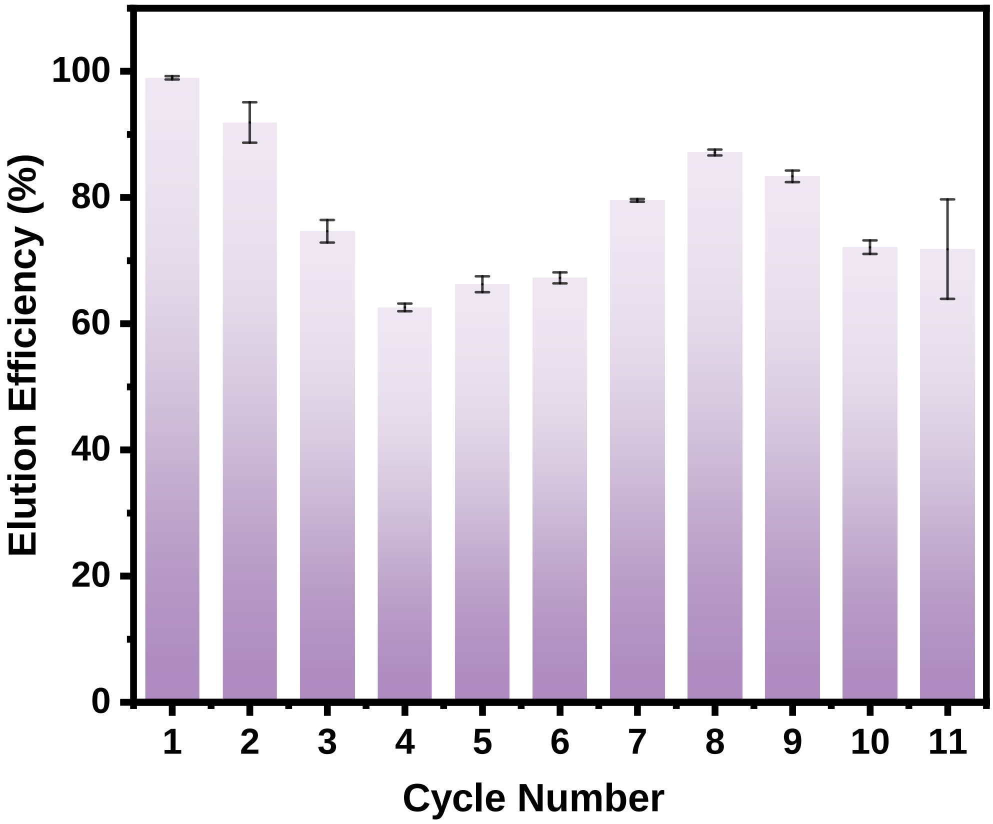

Fig. S22 Elution efficiency of the gold-loaded Ptriaz-CN-A in desorption cycles. Error bars represent standard deviation. n=3 independent experiments. Source data are provided as a Source Data file.

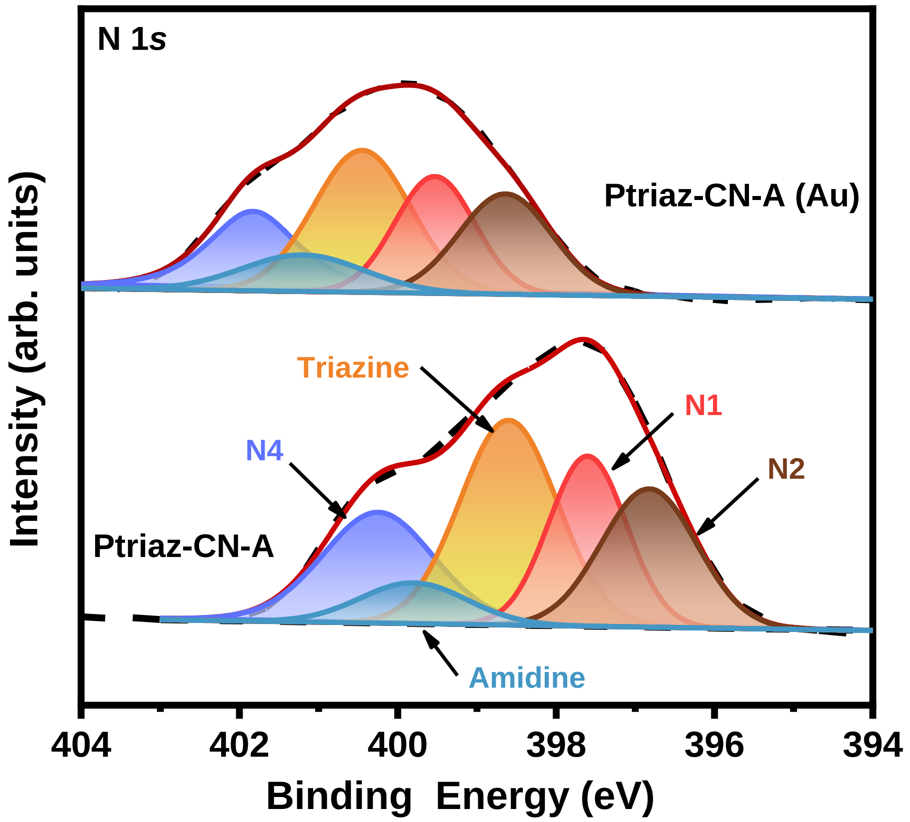

Fig. S23 The N 1*s* XPS spectra of Ptriaz-CN-A before and after Au^{3+} adsorption. The coloured shadings represent the internal integral area of different characteristic peaks. N4 (purple), Amidine (blue), Triazine (orange), N1 (red) and N2 (brown). Source data are provided as a Source Data file.

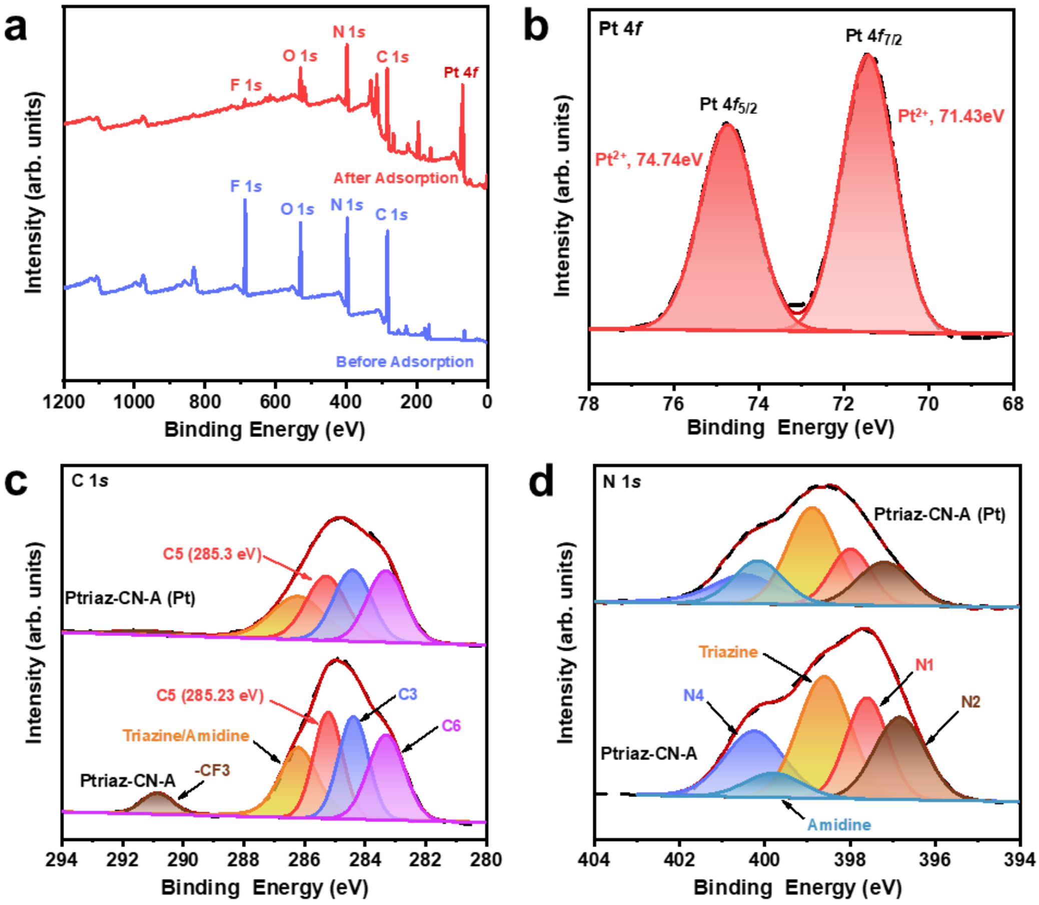

Fig. S24 (a) XPS full spectra of Ptriaz-CN-A before and after Pt^{2+} adsorption. **(b)** High-resolution XPS spectra of Pt 4*f* after Pt^{2+} adsorption. The coloured shadings represent the internal integral area of different characteristic peaks. Pt^{2+} (red). **(c)** The C 1*s* XPS spectra of Ptriaz-CN-A before and after Pt^{2+} adsorption. The coloured shadings represent the internal integral area of different characteristic peaks. C3 (purple), C6 (pink), Triazine/Amidine (orange), C5 (red) and $-CF_3$ (brown). **(d)** The N 1*s* XPS spectra of Ptriaz-CN-A before and after Pt^{2+} adsorption. The coloured shadings represent the internal integral area of different characteristic peaks. N4 (purple), Amidine (blue), Triazine (orange), N1 (red) and N2 (brown). Source data are provided as a Source Data file.

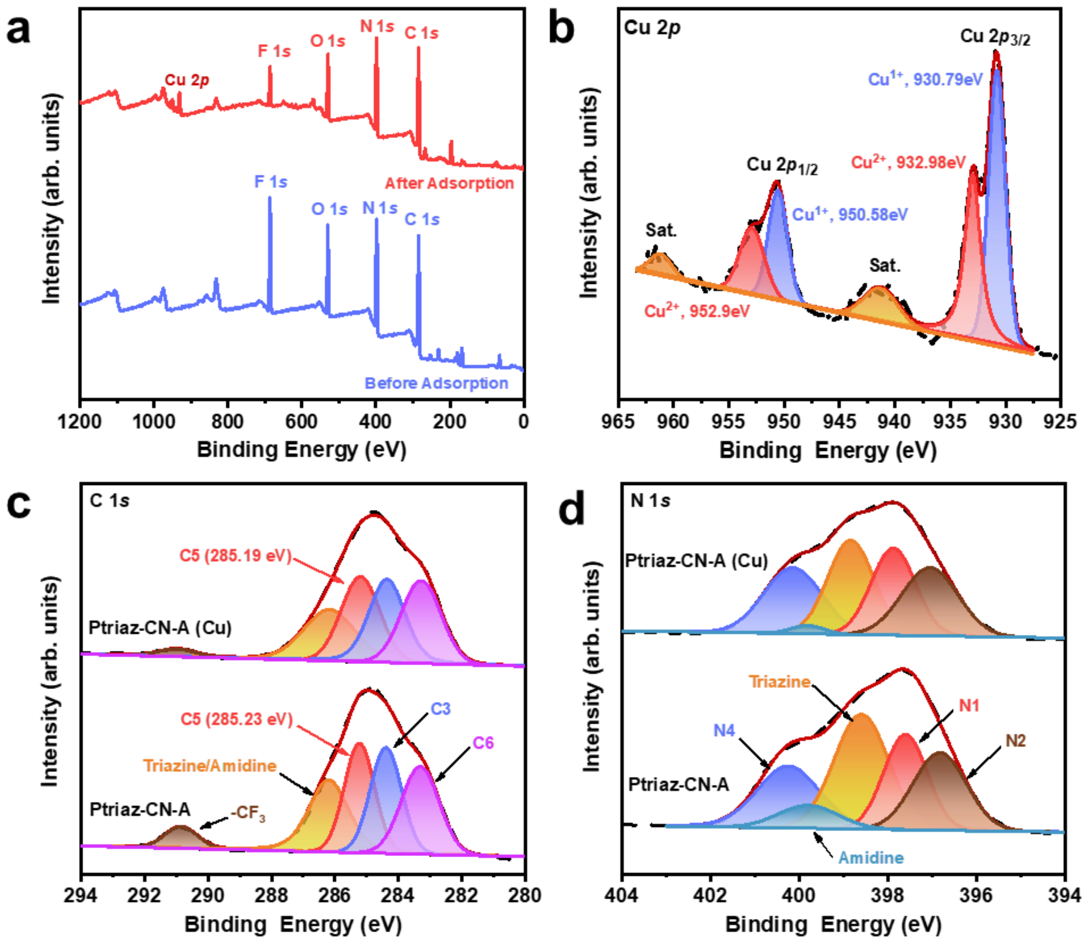

Fig. S25 (a) XPS full spectra of Ptriaz-CN-A before and after Cu^{2+} adsorption. **(b)** High-resolution XPS spectra of Cu 2*p* after Cu^{2+} adsorption. The coloured shadings represent the internal integral area of different characteristic peaks. Cu^{2+} (red) and Cu^{1+}(purple). **(c)** The C 1*s* XPS spectra of Ptriaz-CN-A before and after Cu^{2+} adsorption. The coloured shadings represent the internal integral area of different characteristic peaks. C3 (purple), C6 (pink), Triazine/Amidine (orange), C5 (red) and -CF_3 (brown). **(d)** The N 1*s* XPS spectra of Ptriaz-CN-A before and after Cu^{2+} adsorption. The coloured shadings represent the internal integral area of different characteristic peaks. N4 (purple), Amidine (blue), Triazine (orange), N1 (red) and N2 (brown). Source data are provided as a Source Data file.

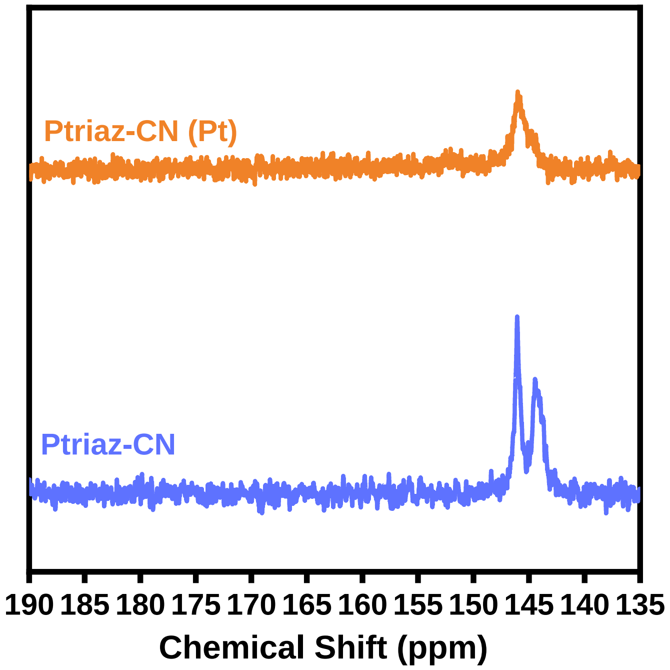

Fig. S26 ^{13}C NMR spectra of Ptriaz-CN and Ptriaz-CN (Pt). Source data are provided as a Source Data file.

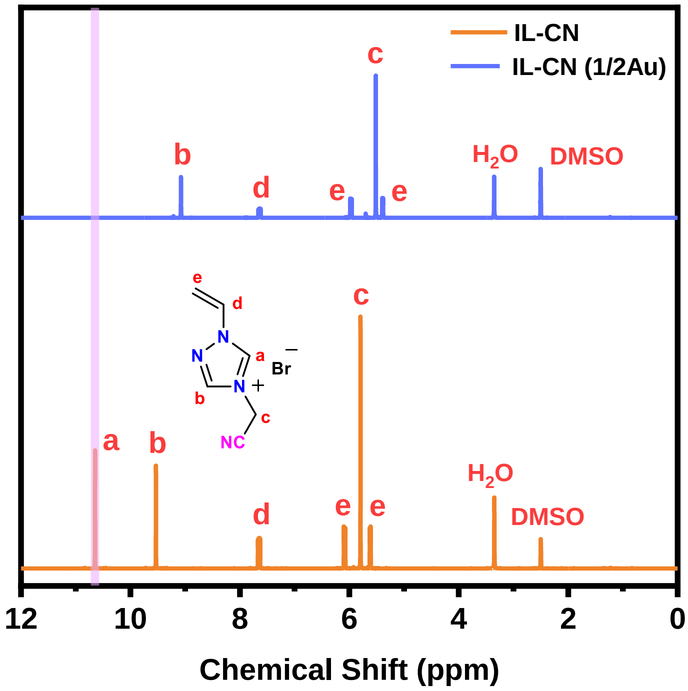

Fig. S27 ^{1}H NMR spectra of ionic liquids monomer 4-cyanomethy-1-viny-1,2,4-triazolium bromide (IL-CN) and IL-CN (1/2Au). Source data are provided as a Source Data file.

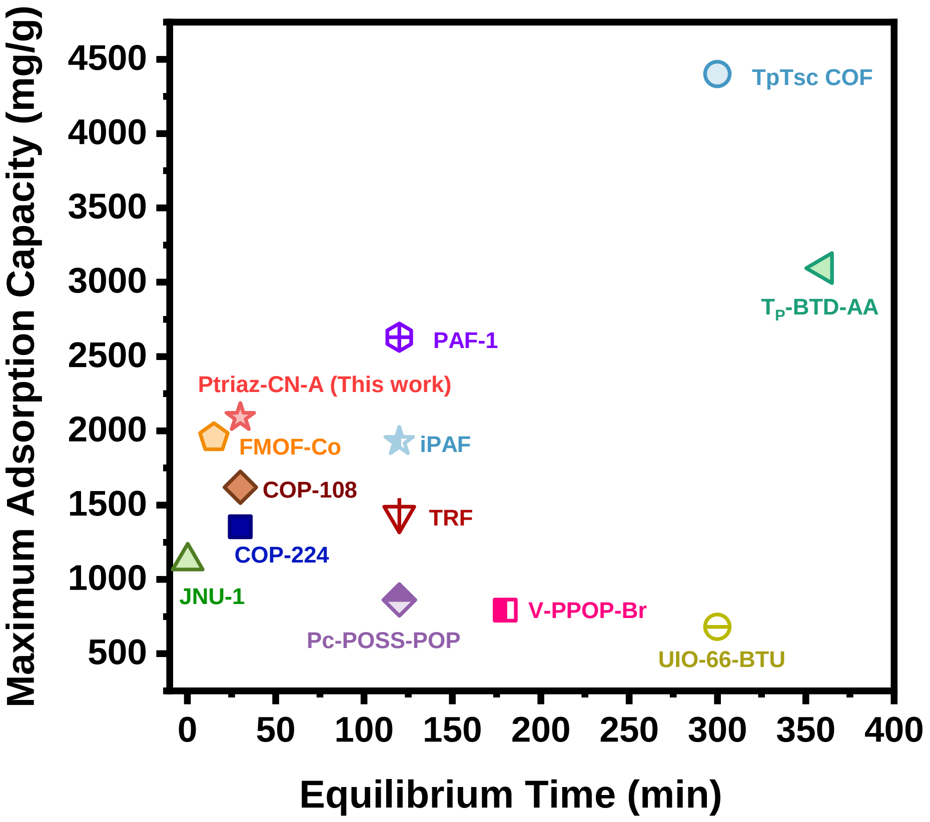

Fig. S28 Comparison of maximum adsorption capacity and equilibrium adsorption time of Ptriaz-CN-A with adsorbents reported in the literature. Source data are provided as a Source Data file.

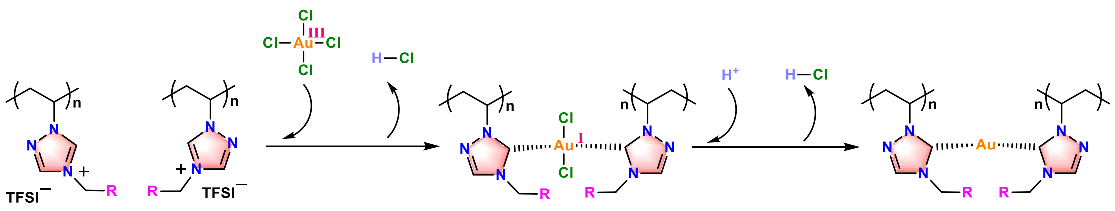

Fig. S29 Bigger geometry for Fig. 5a in main text.

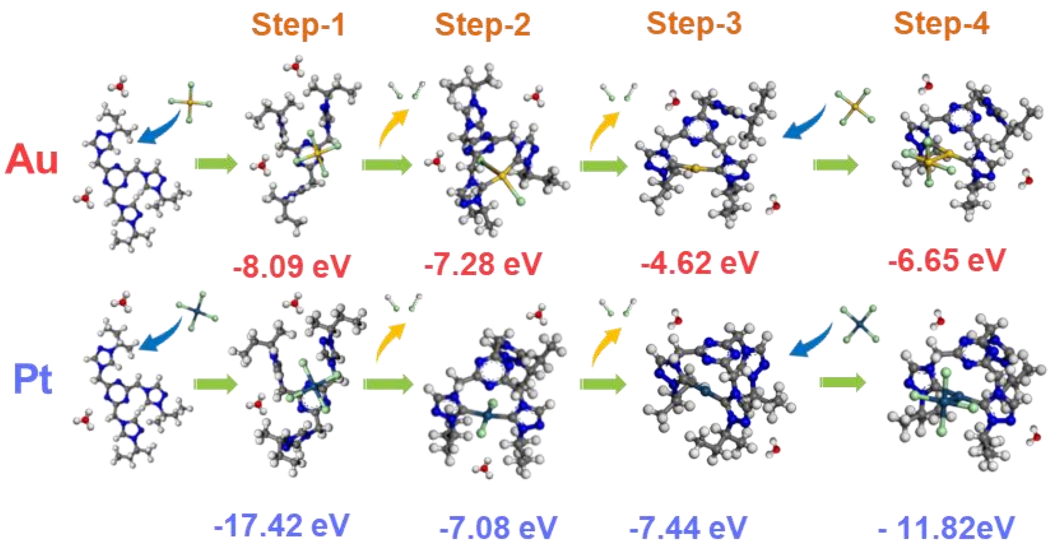

Fig. S30 Bigger geometry for Fig. 5b in main text.

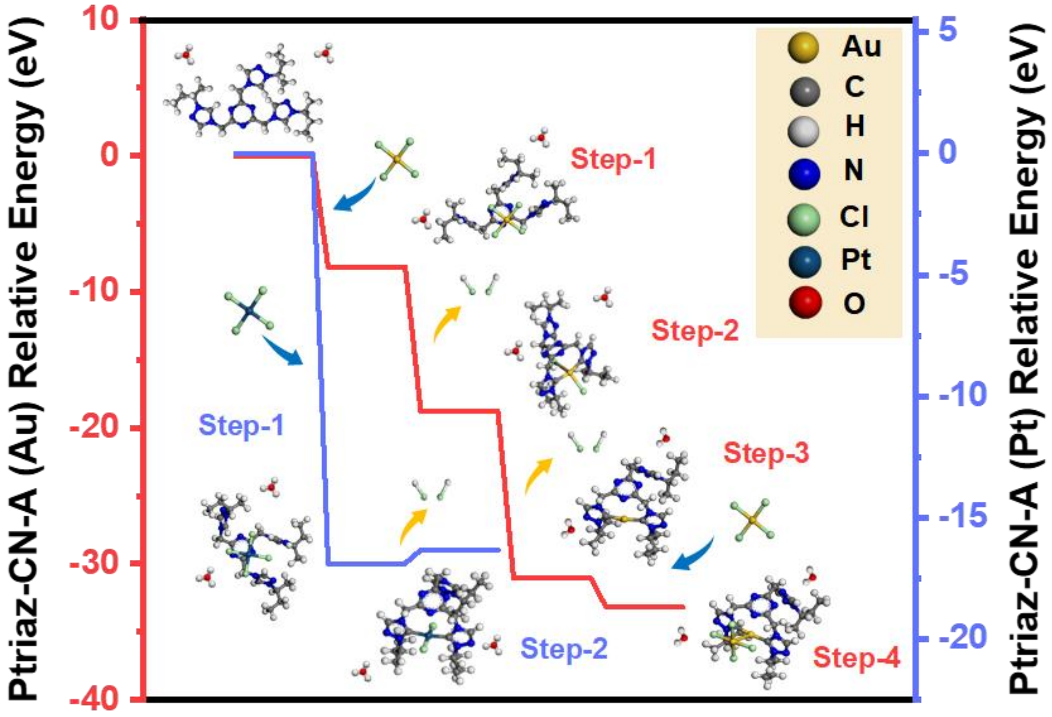

Fig. S31 Bigger geometry for Fig. 5c in main text.

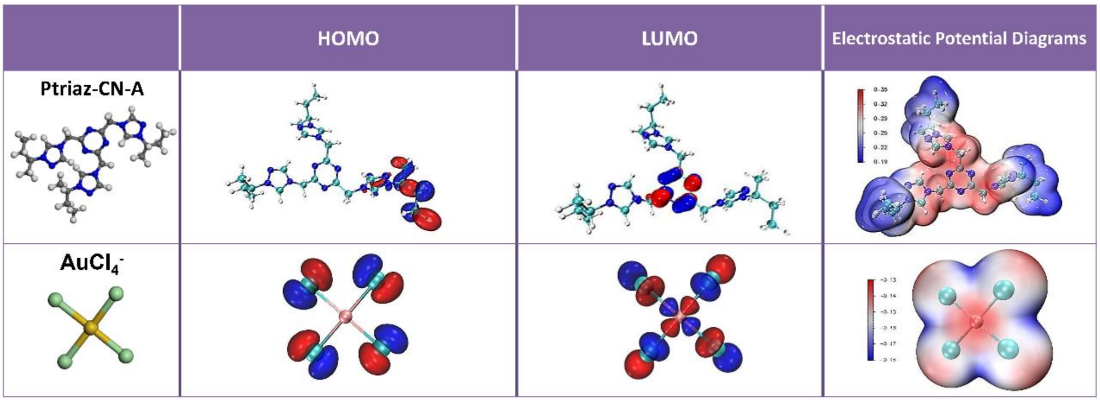

Fig. S32 HOMO and LUMO representations and electrostatic potential diagrams of Ptriaz-CN-A model unit and $AuCl_4^-$.

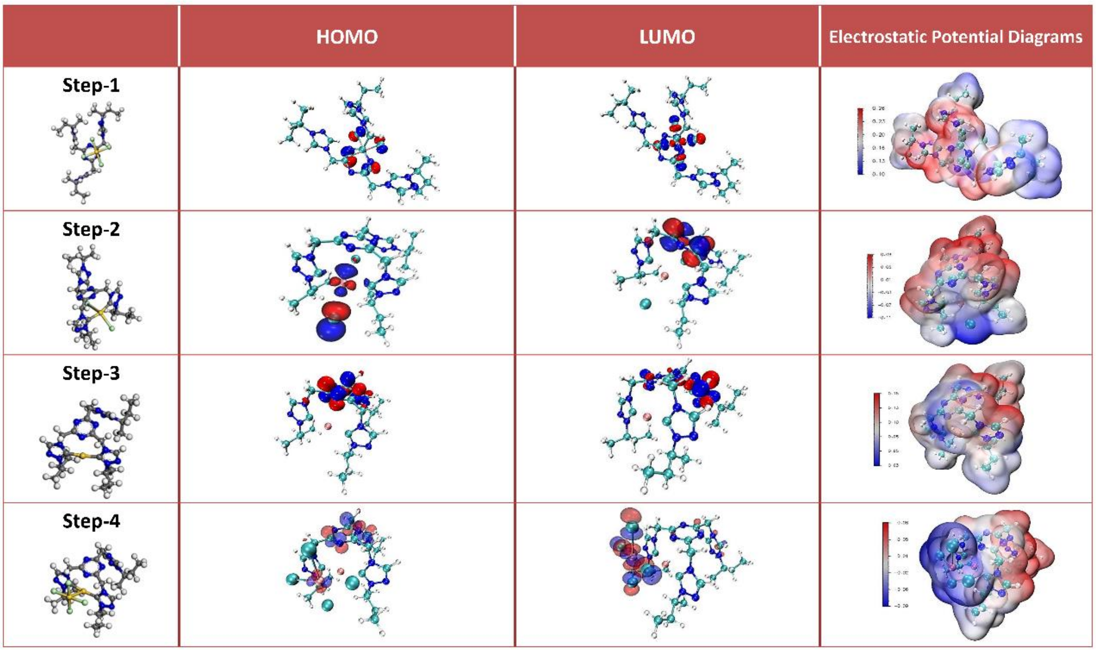

Fig. S33 HOMO and LUMO representations and electrostatic potential diagrams in adsorption process of $AuCl_4^-$ on a model unit of Ptriaz-CN-A.

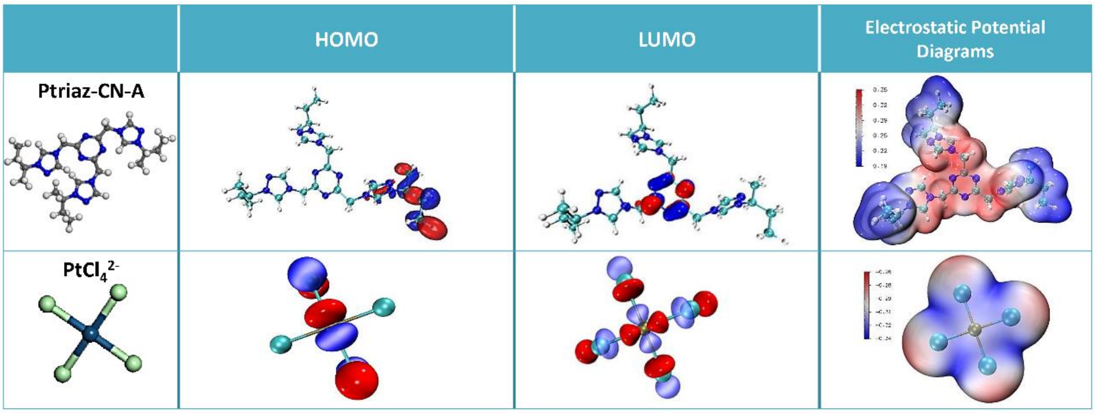

Fig. S34 HOMO and LUMO representations and electrostatic potential diagrams of Ptriaz-CN-A model unit and $PtCl_4^{2-}$.

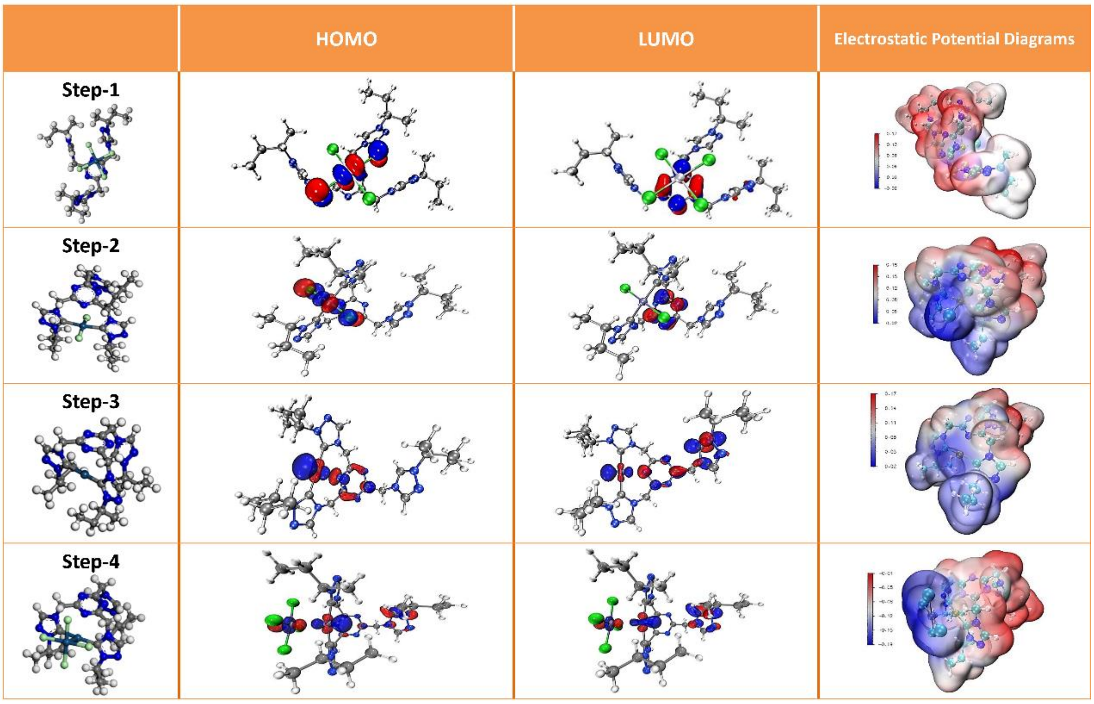

Fig. S35 HOMO and LUMO representations and electrostatic potential diagrams in adsorption process of $PtCl_4^{2-}$ on a model unit of Ptriaz-CN-A.

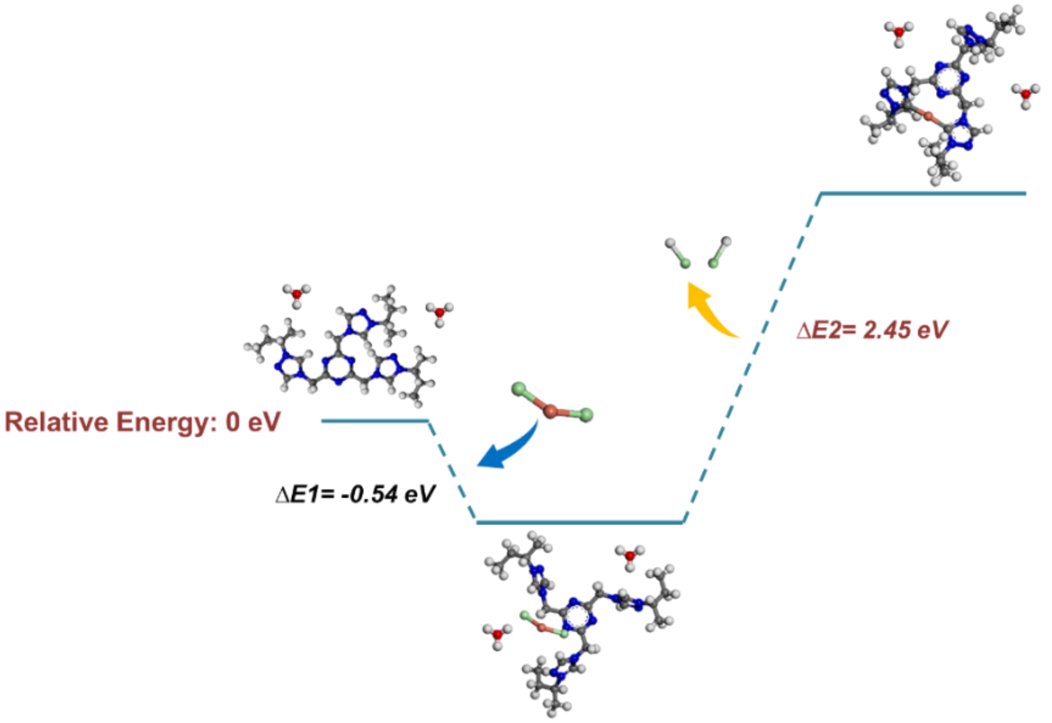

Fig. S36 DFT calculated energy differences in adsorption process of $CuCl_2$ on a model unit of Ptriaz-CN-A.

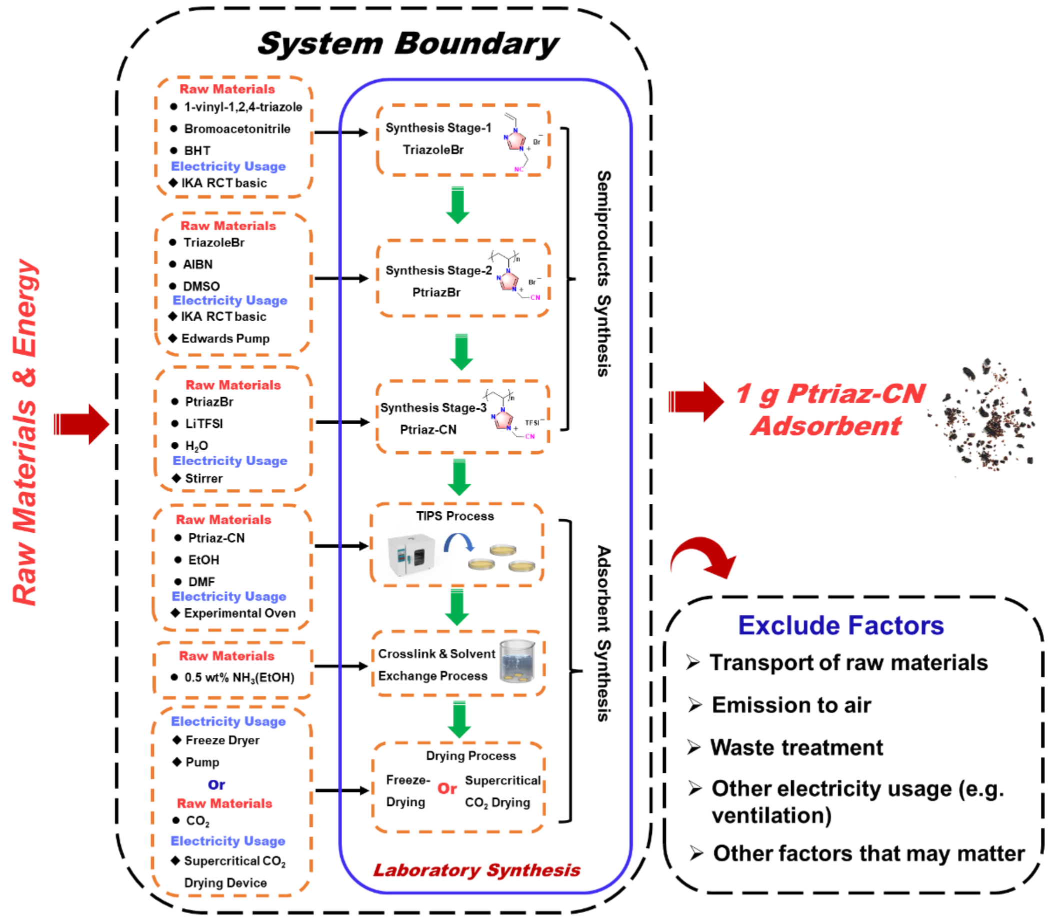

Fig. S37 System boundary for Ptriaz-CN-A production in life cycle assessment.

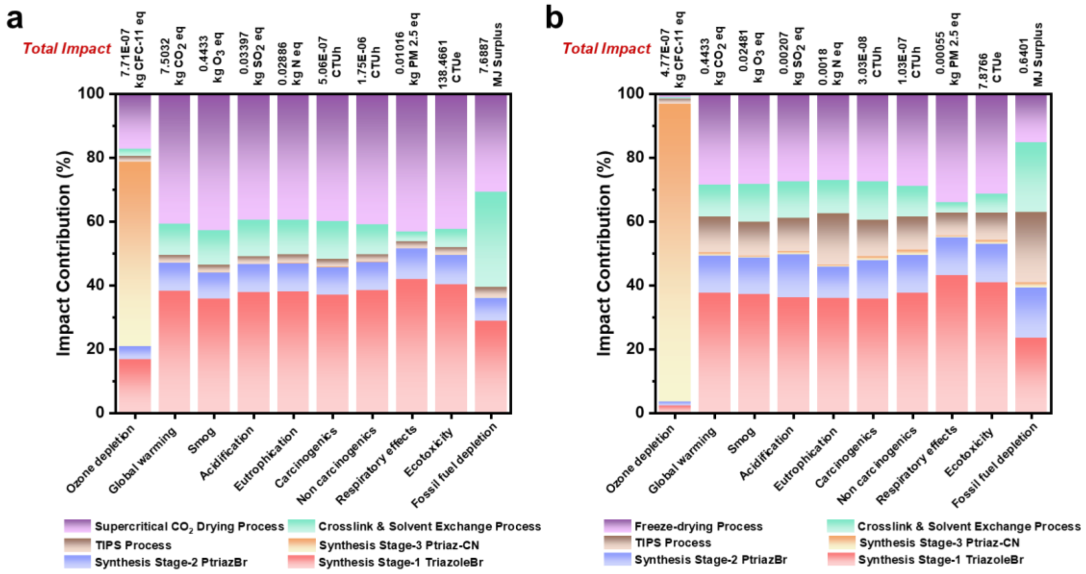

Fig. S38 Contribution of life cycle phases to environmental impacts for 1 g Ptriaz-CN-A production. **a** Scale-1. **b** Scale-2. Source data are provided as a Source Data file.

Supplementary Tables

Table S1 Comparison of **maximum adsorption capacity** for Au^{3+}

Material	*Adsorbent Dose(mg)*	*Au^{3+} Initial Conc. (ppm)*	*Adsorption Time(h)*	*Q_{max} (mg/g)*	*Ref.*
TpTsc COF	5	100~1500	48	4400.23	**6**
PAF-1	5	20~500	48	2629.87	**7**
Tp-BTD-AA	2	25~1200	6	3094.6 Note: under irradiation with 460 nm	**8**
Ptriaz-CN-A	5	100~5000	24	**2090**	**This work**
FMOF-Co	0.5	100	8	1953.7	**9**
iPAF	5	20~500	48	1927.3	**10**
COP-108	100	20~5000	60	1620	**11**
TRF	5	100~600	/	1432	**12**
COP-224	10	30~2000	48	1354	**13**
JNU-1	5	72~2030	/	1124	**14**
Pc−POSS−POP	2	50~500	24	862.07	**15**
V-PPOP-Br	9	180~500	/	792.22	**16**
UiO-66-BTU	10	200~700	6	680.2	**17**

Table S2 Comparison of the **equilibrium time** for Au^{3+}

Material	*Adsorbent Dose(mg)*	*Au^{3+} Initial Conc. (ppm)*	*Contact Time(h)*	*Equilibrium Time(min)*	*Ref.*
TpTsc COF	5	300	48	300	**6**
PAF-1	50	100	48	120	**7**
Tp-BTD-AA	2	100	6	360	**8**
Ptriaz-CN-A	5	100	24	30	**This work**
FMOF-Co	1	100	16	15	**9**
iPAF	50	100	48	120	**10**
COP-108	10	0.1	24	30	**11**
TRF	5	100	7	120	**12**
COP-224	10	0.05	24	30	**13**
JNU-1	5	396	2	0.17	**14**
Pc−POSS−POP	9	100	24	120	**15**
V-PPOP-Br	9	155	5	180	**16**
UiO-66-BTU	10	400	24	300	**17**

Table S3 The K_d values of multiple metal ions in simulated e-waste solution.

	Coexisting Ions							
	**Au^{3+}**	Pt^{2+}	Cu^{2+}	Ni^{2+}	Zn^{2+}	Mg^{2+}	Co^{2+}	Cr^{2+}
K_d	**1011.582**	174.772	0.0948	0.4564	0.0882	0.548	0.0357	1.712

Table S4 Metal content of authentic electronic wastewater (duplicated test).

**Element**	**Test-1 Concentration (ppm)**	**Test-2 Concentration (ppm)**
Cu	346.293	357.56
Ni	58.31	85.32
Pt	5.765	0.534
Au	1.585	2.235
Zn	1.552	1.832

Table S5 Critical values for $AuCl_4^-$ adsorption process in DFT calculations.

**Au**	**System Charge**	**Spin Multiplicity**	**HOMO (a.u.)**	**LUMO (a.u.)**	**HOMO-LUMO gap (kcal/mol)**	**Hardness (kcal/mol)**	**Chemical potential**	**Electron-negativity**
Step 1	2	1	-0.25949	-0.15641	64.684	64.684	-130.491	130.491
Step 2	0	1	-0.13501	-0.08743	29.857	29.857	-69.792	69.792
Step 3	1	2				105.778	-111.837	111.837
Step 4	0	2				82.787	-81.425	81.425

Table S6 Critical values for $PtCl_4^{2-}$ adsorption process in DFT calculations.

Pt	System Charge	Spin Multiplicity	HOMO (a.u.)	LUMO (a.u.)	HOMO-LUMO gap (kcal/mol)	Hardness (kcal/mol)	Chemical potential	Electron-negativity
Step 1	1	1	-0.25419	-0.18324	44.522	44.522	-137.246	137.246
Step 2	1	1	-0.28214	-0.19021	57.687	57.687	-148.202	148.202
Step 3	1	1	-0.21067	-0.18662	15.092	15.092	-124.652	124.652
Step 4	-1	1	-0.03108	-0.01902	7.568	7.568	-15.719	15.719

Table S7. The energy values of each model unit during the whole $AuCl_4^-$ adsorption process.

Model unit	Charge	Spin	E-A.U. (No Solvent)	E-A.U. (Solvent: water)
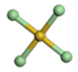	-1	1	-1976.82	-1976.89
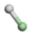	0	1	-460.83	-460.84
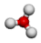	1	1	-76.73	-76.89
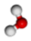	0	1	-76.46	-76.47
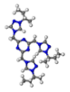	3	1	-1594.84	-1595.30
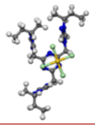	2	1	-3571.96	-3572.20
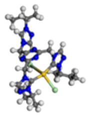	0	1	-2650.69	-2650.77
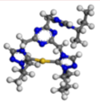	1	2	-1730.02	-1730.10
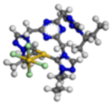	0	2	-3706.92	-3707.02

Table S8. The energy difference of $AuCl_4^-$ adsorption process before/after solvent effect.

	ΔE1 (eV)	ΔE2 (eV)	ΔE3 (eV)	ΔE4 (eV)
No Solvent	-8.16	-10.61	-12.25	-2.18
Solvent Effect	-0.27	-6.80	-4.63	-0.82

Table S9. The energy values of each model unit during the whole $PtCl_4^{2-}$ adsorption process.

Model unit	Charge	Spin	E-A.U. (No Solvent)	E-A.U. (Solvent: water)
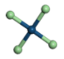	-2	1	-1960.44	-1960.72
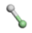	0	1	-460.83	-460.84
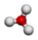	1	1	-76.73	-76.89
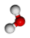	0	1	-76.46	-76.47
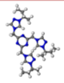	3	1	-1594.84	-1595.30
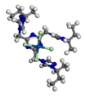	1	1	-3555.90	-3556.12
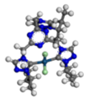	1	1	-2634.22	-2634.32

Table S10. The energy difference of $PtCl_4^{2-}$adsorption process before/after solvent effect.

	ΔE1 (eV)	ΔE2 (eV)
No Solvent	-16.87	0.54
Solvent Effect	-2.72	3.27

Table S11. Eco-inventories used in LCI for 1 kg 1-vinyl-1,2,4-triazole production (The substance was synthesized by a chemical formula).

Input	Amount	Units	Corresponding LCI
Synthesis of 1 kg 1-vinyl-1,2,4-triazole			
Cyclic N-compound	0.7263	kg	Cyclic N-compound {GLO}| market for | APOS, U
Vinyl acetate	0.9052	kg	Vinyl acetate {GLO}| market for | APOS, U

Table S12. Eco-inventories used in LCI for 1 kg bromoacetonitrile production (The substance was synthesized by a chemical formula).

Input	Amount	Units	Corresponding LCI
Synthesis of 1 kg bromoacetonitrile			
Acetonitrile	0.3534	kg	Acetonitrile {GLO}| market for | APOS, U
Acetone	1.7668	kg	Acetone, liquid {RoW}| market for acetone, liquid | APOS, U

Table S13. Eco-inventories used in LCI for 1 kg LiTFSI production (The substance was synthesized by a chemical formula).

Input	Amount	Units	Corresponding LCI
Synthesis of 1 kg LiTFSI			
Trifluoromethane	0.2439	kg	Trifluoromethane {GLO}| market for | APOS, U
Lithium brine	0.0765	kg	Lithium brine, 6.7 % Li {GLO}| market for | APOS, U
lithium bis(fluorosulfonyl) imid	0.6516	kg	Chemical, organic{GLO}| market for | APOS, U

Table S14. Eco-inventories used in LCI for 1 g Ptriaz-CN-A production in **Scale-1** (supercritical CO_2 drying was chosen as drying method).

Input	Amount	Units/ FU	Corresponding LCI
Synthesis of TriazoleBr			
1-vinyl-1,2,4-triazole	0.5124	g	/
bromoacetonitrile	0.9698	g	/
BHT	4.6581E-03	g	Chemical, organic {GLO}| market for | APOS, U
Electricity (for IKA RCT basic)	4.0246	kWh	Electricity, low voltage {GLO}| market group for | APOS, U
Synthesis of PtriazBr			
AIBN	0.01258	g	Chemical, organic {GLO}| market for | APOS, U

DMSO	10.2478	g	Dimethyl sulfoxide {GLO}| market for | APOS, U
Electricity (for Edwards Pump)	0.0210	kWh	Electricity, low voltage {GLO}| market group for | APOS, U
Electricity (for IKA RCT basic)	0.8944	kWh	Electricity, low voltage {GLO}| market group for | APOS, U
Synthesis of Ptriaz-CN			
LiTFSI	1.4114	g	
Water	46.5810	g	Water, deionised {RoW}| market for water, deionised | APOS, U
Electricity (for Stirrer)	4.4718E-03	kWh	Electricity, low voltage {GLO}| market group for | APOS, U
TIPS Process			
EtoH	2.2333	g	Ethanol, without water, in 99.7% solution state, from ethylene {RoW}| market for ethanol, without water, in 99.7% solution state, from ethylene | APOS, U
DMF	13.1167	g	N,N-dimethylformamide {GLO}| market for | APOS, U
Electricity (for Experiment Oven)	0.1975	kWh	Electricity, low voltage {GLO}| market group for | APOS, U
Crosslink & Solvent Change Process			
NH_3	1.9822	g	Ammonia, anhydrous, liquid {RoW}| market for ammonia, anhydrous, liquid | APOS, U
EtOH	394.4622	g	Ethanol, without water, in 99.7% solution state, from ethylene {RoW}| market for ethanol, without water, in 99.7% solution state, from ethylene | APOS, U
Drying Process-Supercritical CO_2 Drying			
Electricity (for instrument)	4.0000	kWh	Electricity, low voltage {GLO}| market group for | APOS, U
CO_2	275.0037	g	Carbon dioxide, liquid {RoW} market for | APOS, U

Table S15. Eco-inventories used in LCI for 1 g Ptriaz-CN-A production in **Scale-2** (freeze-drying was chosen as drying method).

Input	Amount	Units/ FU	Eco-inventory from Ecoinvent v3.8 database
Synthesis of TriazoleBr			
1-vinyl-1,2,4-triazole	0.5059	g	/
bromoacetonitrile	0.9562	g	/
BHT	4.6747E-03	g	Chemical, organic {GLO}| market for | APOS, U

Electricity (for IKA RCT basic)	0.2186	kWh	Electricity, low voltage {GLO}| market group for | APOS, U
Synthesis of PtriazBr			
AIBN	0.0124	g	Chemical, organic {GLO}| market for | APOS, U
DMSO	12.0763	g	Dimethyl sulfoxide {GLO}| market for | APOS, U
Electricity (for Edwards Pump)	1.1383E-03	kWh	Electricity, low voltage {GLO}| market group for | APOS, U
Electricity (for IKA RCT basic)	0.04857	kWh	Electricity, low voltage {GLO}| market group for | APOS, U
Synthesis of Ptriaz-CN			
LiTFSI	1.4113	g	
Water	11.4490	g	Water, deionised {RoW}| market for water, deionised | APOS, U
Electricity (for Stirrer)	2.4284E-04	kWh	Electricity, low voltage {GLO}| market group for | APOS, U
TIPS Process			
EtOH	2.2363	g	Ethanol, without water, in 99.7% solution state, from ethylene {RoW}| market for ethanol, without water, in 99.7% solution state, from ethylene | APOS, U
DMF	13.1235	g	N, N-dimethylformamide {GLO}| market for | APOS, U
Electricity (for Experiment Oven)	0.01073	kWh	Electricity, low voltage {GLO}| market group for | APOS, U
Crosslink & Solvent Change Process			
NH_3	0.1208	g	Ammonia, anhydrous, liquid {RoW}| market for ammonia, anhydrous, liquid | APOS, U
EtOH	23.9483	g	Ethanol, without water, in 99.7% solution state, from ethylene {RoW}| market for ethanol, without water, in 99.7% solution state, from ethylene | APOS, U
Drying Process-Freezing Drying			
Electricity (for Freeze Dryer)	0.1336	kWh	Electricity, low voltage {GLO}| market group for | APOS, U
Electricity (for Pump)	0.04493	kWh	Electricity, low voltage {GLO}| market group for | APOS, U

Table S16 Environmental impacts at each stage of Ptriaz-CN-A production in Scale-1.

Impact category	Unit	Total Scale-1	Synthesis Stage-1	Synthesis Stage-2	Synthesis Stage-3	TIPS Process	Crosslink &Solvent exchange	Drying Process
OD	kg CFC -11 eq	7.71E-07	1.29E-07	3.12E-08	4.47E-07	1.37E-08	1.78E-08	1.33E-07
GW	kg CO_2 eq	7.503232	2.86E+00	6.62E-01	7.53E-03	1.82E-01	7.27E-01	3.06E+00
SM	kg O_3 eq	0.443254	1.58E-01	3.66E-02	3.57E-04	9.94E-03	4.87E-02	1.89E-01
AC	kg SO_2 eq	0.033973	1.28E-02	3.01E-03	3.46E-05	8.10E-04	3.90E-03	1.34E-02
ET	kg N eq	0.02886	1.10E-02	2.52E-03	2.21E-05	7.97E-04	3.13E-03	1.14E-02
HHC	CTUh	5.06E-07	1.87E-07	4.36E-08	5.31E-10	1.22E-08	6.02E-08	2.02E-07
HHNC	CTUh	1.75E-06	6.71E-07	1.55E-07	2.35E-09	4.17E-08	1.62E-07	7.18E-07
RE	kg PM2.5 eq	0.010166	4.25E-03	9.77E-04	7.50E-06	2.37E-04	2.95E-04	4.40E-03
EC	CTUe	138.4661	5.57E+01	1.28E+01	1.51E-01	3.26E+00	7.77E+00	5.87E+01
FF	MJ surplus	7.688747	2.22E+00	5.60E-01	1.27E-02	2.42E-01	2.30E+00	2.36E+00

Table S17 Environmental impacts of each substance in Synthesis Stage-1 (Scale-1).

Impact category	Unit	Synthesis Stage-1	1-vinyl-1,2,4-triazole	bromoaceto nitrile	BHT	Electricity (heating)
OD	kg CFC -11 eq	1.29E-07	4.50E-09	1.49E-10	9.07E-13	1.24E-07
GW	kg CO_2 eq	2.86E+00	0.006643	0.005868	9.04E-06	2.851961
SM	kg O_3 eq	1.58E-01	0.00036	0.000301	4.52E-07	0.15773
AC	kg SO_2 eq	1.28E-02	3.05E-05	2.63E-05	3.58E-08	0.012769
ET	kg N eq	1.10E-02	3.90E-05	1.55E-05	1.64E-08	0.010946
HHC	CTUh	1.87E-07	4.75E-10	2.49E-10	4.90E-13	1.87E-07
HHNC	CTUh	6.71E-07	1.64E-09	6.91E-10	1.31E-12	6.69E-07
RE	kg PM2.5 eq	4.25E-03	5.90E-06	2.66E-06	5.33E-09	0.004244159
EC	CTUe	5.57E+01	0.170061	0.041708	7.73E-05	55.5021
FF	MJ surplus	2.22E+00	0.011605	0.020419	3.69E-05	2.183006

Table S18 Environmental impacts of each substance in Synthesis Stage-2 (Scale-1).

Impact category	Unit	Synthesis Stage-2	AIBN	DMSO	Electricity (pump)	Electricity (heating)
OD	kg CFC -11 eq	3.12E-08	2.45E-12	2.88E-09	6.48E-10	2.76E-08
GW	kg CO_2 eq	6.62E-01	2.44E-05	0.013348	0.014854	0.633769
SM	kg O_3 eq	3.66E-02	1.22E-06	0.000753	0.000822	0.035051
AC	kg SO_2 eq	3.01E-03	9.66E-08	0.000104	6.65E-05	0.002838
ET	kg N eq	2.52E-03	4.42E-08	3.53E-05	5.70E-05	0.002432
HHC	CTUh	4.36E-08	1.32E-12	1.14E-09	9.71E-10	4.14E-08
HHNC	CTUh	1.55E-07	3.53E-12	3.26E-09	3.48E-09	1.49E-07
RE	kg PM2.5 eq	9.77E-04	1.44E-08	1.19E-05	2.21E-05	0.000943
EC	CTUe	1.28E+01	0.000209	0.222544	0.289073	12.333801
FF	MJ surplus	5.60E-01	9.96E-05	0.063376	0.011369824	0.4851125

Table S19 Environmental impacts of each substance in Synthesis Stage-3 (Scale-1).

Impact category	Unit	Synthesis Stage-3	LiTFSI	Water	Electricity (Stirrer)
OD	kg CFC -11 eq	4.47E-07	4.46E-07	7.73E-12	1.38E-10
GW	kg CO_2 eq	7.53E-03	0.004348	1.41E-05	0.003169
SM	kg O_3 eq	3.57E-04	0.000181	8.66E-07	0.000175
AC	kg SO_2 eq	3.46E-05	2.03E-05	1.05E-07	1.42E-05
ET	kg N eq	2.21E-05	9.91E-06	5.38E-08	1.22E-05
HHC	CTUh	5.31E-10	3.21E-10	2.64E-12	2.07E-10
HHNC	CTUh	2.35E-09	1.60E-09	1.11E-11	7.43E-10
RE	kg PM2.5 eq	7.50E-06	2.76E-06	1.84E-08	4.72E-06
EC	CTUe	1.51E-01	0.088321	0.000678	0.061669
FF	MJ surplus	1.27E-02	0.010286	1.62E-05	0.002426

Table S20 Environmental impacts of each substance in TIPS process (Scale-1).

Impact category	Unit	TIPS process	EtOH	DMF	Electricity (heating)
OD	kg CFC -11 eq	1.37E-08	9.95E-11	7.51E-09	6.10E-09
GW	kg CO_2 eq	1.82E-01	0.0040798	0.0382044	0.1399574
SM	kg O_3 eq	9.94E-03	0.0002743	0.0019203	0.0077405
AC	kg SO_2 eq	8.10E-04	2.20E-05	0.0001615	0.0006266
ET	kg N eq	7.97E-04	1.76E-05	0.0002424	0.0005372
HHC	CTUh	1.22E-08	3.39E-10	2.66E-09	9.15E-09
HHNC	CTUh	4.17E-08	9.10E-10	8.00E-09	3.28E-08
RE	kg PM2.5 eq	2.37E-04	1.66E-06	2.68E-05	0.0002083
EC	CTUe	3.26E+00	0.0437756	0.488294	2.723714
FF	MJ surplus	2.42E-01	0.0129843	0.122344	0.107129

Table S21 Environmental impacts of each substance in Crosslink & Solvent exchange process (Scale-1).

Impact category	Unit	Crosslink & Solvent exchange	Ammonia	EtOH
OD	kg CFC -11 eq	1.78E-08	1.73E-10	1.76E-08
GW	kg CO_2 eq	7.27E-01	0.0064504	0.720594
SM	kg O_3 eq	4.87E-02	0.00020734	0.0484475
AC	kg SO_2 eq	3.90E-03	1.70E-05	0.0038782
ET	kg N eq	3.13E-03	1.19E-05	0.0031167
HHC	CTUh	6.02E-08	2.94E-10	5.99E-08
HHNC	CTUh	1.62E-07	8.85E-10	1.61E-07
RE	kg PM2.5 eq	2.95E-04	1.83E-06	0.0002929
EC	CTUe	7.77E+00	0.0411422	7.731867
FF	MJ surplus	2.30E+00	0.0083758	2.293353

Table S22 Environmental impacts of each substance in Supercritical CO_2 drying process (Scale-1).

Impact category	Unit	Supercritical CO_2 drying	Electricity (for instrument)	CO_2
OD	kg CFC -11 eq	1.33E-07	1.24E-07	8.99E-09
GW	kg CO_2 eq	3.06E+00	2.834533	0.225404
SM	kg O_3 eq	1.89E-01	0.156766	0.0325217
AC	kg SO_2 eq	1.34E-02	0.0126911	0.0007082
ET	kg N eq	1.14E-02	0.0108791	0.0005081
HHC	CTUh	2.02E-07	1.85E-07	1.66E-08
HHNC	CTUh	7.18E-07	6.65E-07	5.30E-08
RE	kg PM2.5 eq	4.40E-03	0.004218	0.0001784
EC	CTUe	5.87E+01	55.162925	3.564127
FF	MJ surplus	2.36E+00	2.169666	0.187144

Table S23 Environmental impacts at each stage of Ptriaz-CN-A production in Scale-2.

Impact category	Unit	Total Scale-2	Synthesis Stage-1	Synthesis Stage-2	Synthesis Stage-3	TIPS Process	Crosslink &Solvent exchange	Drying Process
OD	kg CFC -11 eq	4.77E-07	1.13E-08	4.94E-09	4.46E-07	7.95E-09	1.08E-09	5.51E-09
GW	kg CO_2 eq	0.443264	0.167231	5.10E-02	4.52E-03	4.99E-02	4.41E-02	1.26E-01
SM	kg O_3 eq	0.024811	0.009218	2.84E-03	1.91E-04	2.62E-03	2.95E-03	7.00E-03
AC	kg SO_2 eq	0.002071	7.50E-04	2.80E-04	2.11E-05	2.18E-04	2.36E-04	5.66E-04
ET	kg N eq	0.001800	6.48E-04	1.77E-04	1.06E-05	2.89E-04	1.90E-04	4.85E-04
HHC	CTUh	3.03E-08	1.08E-08	3.65E-09	3.33E-10	3.50E-09	3.66E-09	8.27E-09
HHNC	CTUh	1.03E-07	3.86E-08	1.21E-08	1.64E-09	1.07E-08	9.81E-09	2.97E-08
RE	kg PM2.5 eq	0.000554	2.39E-04	6.65E-05	3.02E-06	3.98E-05	1.79E-05	1.88E-04
EC	CTUe	7.876643	3.22E+00	9.48E-01	9.18E-02	6.80E-01	4.72E-01	2.46E+00
FF	MJ surplus	0.640123	1.50E-01	1.02E-01	1.04E-02	1.41E-01	1.40E-01	9.68E-02

Table S24 Environmental impacts of each substance in Synthesis Stage-1 (Scale-2).

Impact category	Unit	Synthesis Stage-1	1-vinyl-1,2,4-triazole	bromoaceto nitrile	BHT	Electricity (heating)
OD	kg CFC -11 eq	1.13E-08	4.44E-09	1.47E-10	9.10E-13	6.75E-09
GW	kg CO_2 eq	0.167231	0.006559	0.005786	9.07E-06	0.154877
SM	kg O_3 eq	0.009218	0.000356	0.000296	4.53E-07	0.008566
AC	kg SO_2 eq	7.50E-04	3.01E-05	2.60E-05	3.59E-08	0.000693
ET	kg N eq	6.48E-04	3.85E-05	1.53E-05	1.64E-08	0.000594
HHC	CTUh	1.08E-08	4.69E-10	2.45E-10	4.92E-13	1.01E-08
HHNC	CTUh	3.86E-08	1.62E-09	6.81E-10	1.31E-12	3.63E-08
RE	kg PM2.5 eq	2.39E-04	5.83E-06	2.62E-06	5.35E-09	0.0002304
EC	CTUe	3.22E+00	0.167913	0.041122	7.76E-05	3.014063
FF	MJ surplus	1.50E-01	0.011458	0.020132	3.70E-05	0.118549

Table S25 Environmental impacts of each substance in Synthesis Stage-2 (Scale-2).

Impact category	Unit	Synthesis Stage-2	AIBN	DMSO	Electricity (pump)	Electricity (heating)
OD	kg CFC -11 eq	4.94E-09	2.41E-12	3.40E-09	3.52E-11	1.50E-09
GW	kg CO_2 eq	5.10E-02	2.41E-05	0.015729	0.000807	0.034417
SM	kg O_3 eq	2.84E-03	1.20E-06	0.000887	4.46E-05	0.001903
AC	kg SO_2 eq	2.80E-04	9.52E-08	0.000122	3.61E-06	0.000154
ET	kg N eq	1.77E-04	4.36E-08	4.16E-05	3.10E-06	0.000132
HHC	CTUh	3.65E-09	1.30E-12	1.35E-09	5.28E-11	2.25E-09
HHNC	CTUh	1.21E-08	3.47E-12	3.84E-09	1.89E-10	8.07E-09
RE	kg PM2.5 eq	6.65E-05	1.42E-08	1.41E-05	1.20E-06	5.12E-05
EC	CTUe	9.48E-01	0.000206	0.262252	0.015698	0.669792
FF	MJ surplus	1.02E-01	9.81E-05	0.074683	0.000617	0.026344

Table S26 Environmental impacts of each substance in Synthesis Stage-3 (Scale-2).

Impact category	Unit	Synthesis Stage-3	LiTFSI	Water	Electricity (Stirrer)
OD	kg CFC -11 eq	4.46E-07	4.46E-07	1.90E-12	7.50E-12
GW	kg CO_2 eq	4.52E-03	0.004348	3.47E-06	0.000172
SM	kg O_3 eq	1.91E-04	0.000181	2.13E-07	9.52E-06
AC	kg SO_2 eq	2.11E-05	2.03E-05	2.59E-08	7.70E-07
ET	kg N eq	1.06E-05	9.91E-06	1.32E-08	6.60E-07
HHC	CTUh	3.33E-10	3.21E-10	6.49E-13	1.13E-11
HHNC	CTUh	1.64E-09	1.60E-09	2.72E-12	4.04E-11
RE	kg PM2.5 eq	3.02E-06	2.76E-06	4.53E-09	2.56E-07
EC	CTUe	9.18E-02	0.088315	0.000167	0.003349
FF	MJ surplus	1.04E-02	0.010285	3.99E-06	0.000132

Table S27 Environmental impacts of each substance in TIPS process (Scale-2).

Impact category	Unit	TIPS process	EtOH	DMF	Electricity (heating)
OD	kg CFC -11 eq	7.95E-09	9.97E-11	7.52E-09	3.31E-10
GW	kg CO_2 eq	4.99E-02	0.004085	0.038224	0.0076
SM	kg O_3 eq	2.62E-03	0.000275	0.001921	0.00042
AC	kg SO_2 eq	2.18E-04	2.20E-05	0.000162	3.40E-05
ET	kg N eq	2.89E-04	1.77E-05	0.000242	2.92E-05
HHC	CTUh	3.50E-09	3.40E-10	2.66E-09	4.97E-10
HHNC	CTUh	1.07E-08	9.11E-10	8.00E-09	1.78E-09
RE	kg PM2.5 eq	3.98E-05	1.66E-06	2.68E-05	1.13E-05
EC	CTUe	6.80E-01	0.043834	0.48855	0.147912
FF	MJ surplus	1.41E-01	0.013002	0.122408	0.005818

Table S28 Environmental impacts of each substance in Crosslink & Solvent exchange process (Scale-2).

Impact category	Unit	Crosslink & Solvent exchange	Ammonia	EtOH
OD	kg CFC -11 eq	1.08E-09	1.05E-11	1.07E-09
GW	kg CO_2 eq	4.41E-02	0.000392	0.043748
SM	kg O_3 eq	2.95E-03	1.26E-05	0.002941
AC	kg SO_2 eq	2.36E-04	1.03E-06	0.000235
ET	kg N eq	1.90E-04	7.21E-07	0.000189
HHC	CTUh	3.66E-09	1.78E-11	3.64E-09
HHNC	CTUh	9.81E-09	5.37E-11	9.76E-09
RE	kg PM2.5 eq	1.79E-05	1.11E-07	1.78E-05
EC	CTUe	4.72E-01	0.002498	0.469411
FF	MJ surplus	1.40E-01	0.000509	0.139232

Table S29 Environmental impacts of each substance in Freeze-drying process (Scale-2).

Impact category	Unit	Freeze-drying	Electricity (freeze-dryer)	Electricity (pump)
OD	kg CFC -11 eq	5.51E-09	4.13E-09	1.39E-09
GW	kg CO_2 eq	1.26E-01	0.094647	0.031836
SM	kg O_3 eq	7.00E-03	0.005235	0.001761
AC	kg SO_2 eq	5.66E-04	0.000424	0.000143
ET	kg N eq	4.85E-04	0.000363	0.000122
HHC	CTUh	8.27E-09	6.19E-09	2.08E-09
HHNC	CTUh	2.97E-08	2.22E-08	7.47E-09
RE	kg PM2.5 eq	1.88E-04	0.000141	4.74E-05
EC	CTUe	2.46E+00	1.841927	0.619557
FF	MJ surplus	9.68E-02	0.072447	0.024368

Table S30. Consumption for the synthesis of 1 g Ptriaz-CN-A in **Scale-1**. (Laboratory research, small dose feeding, supercritical CO_2 drying as drying method).

Raw Materials	Amount	Units/ FU	Unit price (CNY)	Cost (CNY/g)	Brand
Synthesis of TriazoleBr					
1-vinyl-1,2,4-triazole	0.5124	g	186.169	95.393	Santa Cruz Biotechnology
bromoacetonitrile	0.9698	g	2.463	2.389	Leyan
BHT	4.6581E-03	g	0.200	0.0009	Adamas
Electricity (for IKA RCT basic)	4.0246	kWh	0.617	2.483	/
Synthesis of PtriazBr					
AIBN	0.01258	g	0.53	0.007	Collins
DMSO	10.2478	g	0.284	2.910	Macklin
Electricity (for Edwards Pump)	0.0210	kWh	0.617	0.013	/
Electricity (for IKA RCT basic)	0.8944	kWh	0.617	0.552	/
Synthesis of Ptriaz-CN					
LiTFSI	1.4114	g	4.312	6.086	Macklin
Water	46.5810	g	0.02	0.932	/
Electricity (for Stirrer)	4.4718E-03	kWh	0.617	0.003	/
TIPS Process					
EtOH	2.2333	g	0.006	0.013	Sinopharm Chemical Reagent
DMF	13.1167	g	0.041	0.538	Greagent
Electricity (for Experiment Oven)	0.1975	kWh	0.617	0.122	/
Crosslink & Solvent Change Process					
NH_3	1.9822	g	0.081	0.161	Wendong
EtOH	394.4622	g	0.006	2.367	Sinopharm Chemical Reagent
Drying Process-Supercritical CO_2 Drying					
Electricity (for instrument)	4.0000	kWh	0.617	2.468	/
CO_2	275.0037	g	0.002	0.55	WenDong
**Total Cost (raw materials + electricity): 116.988**					

Note: The average price of electricity in Shanghai is 0.617 CNY/kWh.

Table S31. Consumption for the synthesis of 1 g Ptriaz-CN-A in **Scale-2**. (maximum production scale of the laboratory, freeze-drying as drying method).

Raw Materials	Amount	Units/ FU	Unit price	Cost (CNY/g)	Brand
Synthesis of TriazoleBr					
1-vinyl-1,2,4-triazole	0.5059	g	186.169	94.183	Santa Cruz Biotechnology
bromoacetonitrile	0.9562	g	2.463	2.355	Leyan
BHT	4.6747E-03	g	0.200	0.0009	Adamas
Electricity (for IKA RCT basic)	0.2186	kWh	0.617	0.135	/
Synthesis of PtriazBr					
AIBN	0.0124	g	0.53	0.007	Collins
DMSO	12.0763	g	0.284	3.430	Macklin
Electricity (for Edwards Pump)	1.1383E-03	kWh	0.617	0.0007	/
Electricity (for IKA RCT basic)	0.04857	kWh	0.617	0.030	/
Synthesis of Ptriaz-CN					
LiTFSI	1.4113	g	4.312	6.086	Macklin
Water	11.4490	g	0.02	0.229	/
Electricity (for Stirrer)	2.4284E-04	kWh	0.617	0.0001	/
TIPS Process					
EtOH	2.2363	g	0.006	0.0134	Sinopharm Chemical Reagent
DMF	13.1235	g	0.041	0.538	Greagent
Electricity (for Experiment Oven)	0.01073	kWh	0.617	0.007	/
Crosslink & Solvent Change Process					
NH_3	0.1208	g	0.081	0.01	WenDong
EtOH	23.9483	g	0.006	0.144	Sinopharm Chemical Reagent
Drying Process-Freeze-Drying					
Electricity (for freeze dryer)	0.1336	kWh	0.617	0.082	/
Electricity (for pump)	0.04493	kWh	0.617	0.028	/
**Total Cost (raw materials + electricity): 107.280**					

Note: The average price of electricity in Shanghai is 0.617 CNY/kWh.

Table S32. Preparation method and price of 1-vinylimidazole and 1-vinyl-1,2,4-triazole.

	**Reactions**	**Product**
**1-Vinylimidazole**	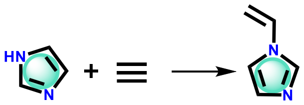<br>**Imidazole**: 160 CNY/kg<br>**Acetylene**: 38 CNY/kg	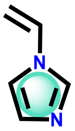<br>640 CNY/kg
**1-Vinyl-1,2,4-Triazole**	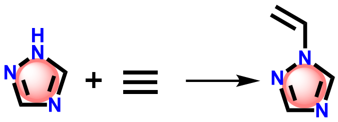<br>**1,2,4-Triazole**: 219 CNY/kg<br>**Acetylene**: 38 CNY/kg	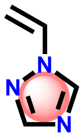<br>186 CNY/g<br>(**The cheapest price online**)

Supplementary References

1. Frisch, M. et al. Gaussian 09 (Revision D.01). (2009).
2. Zhang, J. & Lu, T. Efficient evaluation of electrostatic potential with computerized optimized code. *Phys. Chem. Chem. Phys.* **23**, 20323-20328 (2021).
3. Lu, T. & Chen, F. Multiwfn: a multifunctional wavefunction analyzer. *J. Comput. Chem.* **33**, 580-592 (2012).
4. Xiong, L. & Pei, Y. Symmetric growth of dual-packed kernel: exploration of the evolution of $Au_{40}(SR)_{24}$ to $Au_{49}(SR)_{27}$ and $Au_{58}(SR)_{30}$ clusters via the $2e^-$-reduction cluster growth mechanism. *ACS Omega* **6**, 18024-18032 (2021).
5. Pei, Y. et al. Interlocked catenane-like structure predicted in $Au_{24}(SR)_{20}$: implication to structural evolution of thiolated gold clusters from homoleptic gold(I) thiolates to core-stacked nanoparticles. *J. Am. Chem. Soc.* **134**, 3015-3024 (2012).
6. Zhang, L. et al. Covalent organic frameworks constructed by flexible alkyl amines for efficient gold recovery from leaching solution of e-waste. *Chem. Eng. J.* **426**, 131865 (2021).
7. Ma, T. et al. Efficient gold recovery from e-waste via a chelate-containing porous aromatic framework. *ACS Appl. Mater. Interfaces* **12**, 30474-30482 (2020).
8. Yang, S. et al. Covalent organic framework isomers for photoenhanced gold recovery from e-waste with high efficiency and selectivity. *ACS Sustainable Chem. Eng.* **10**, 9719-9731 (2022).
9. Liu, J., Deng, Z., Yu, H. & Wang, L. Ferrocene-based metal-organic framework for highly efficient recovery of gold from WEEE. *Chem. Eng. J.* **410**, 128360

(2021).

10. Ma, T. et al. Turning electronic waste to continuous-flow reactor using porous aromatic frameworks. *ACS Appl. Mater. Interfaces* **14**, 25601-25608 (2022).

11. Hong, Y. et al. Precious metal recovery from electronic waste by a porous porphyrin polymer. *Proc. Natl. Acad. Sci. U.S.A.* **117**, 16174-16180 (2020).

12. Chen, X., Xiang, Y., Xu, L. & Liu, G. Recovery and reduction of Au(III) from mixed metal solution by thiourea-resorcinol-formaldehyde microspheres. *J. Hazard. Mater.* **397**, 122812 (2020).

13. Nguyen, T. S., Hong, Y., Dogan, N. A. & Yavuz, C. T. Gold Recovery from E-Waste by Porous Porphyrin–Phenazine Network Polymers. *Chem. Mater.* **32**, 5343-5349 (2020).

14. Qian, H. L., Meng, F. L., Yang, C. X. & Yan, X. P. Irreversible amide-linked covalent organic framework for selective and ultrafast gold recovery. *Angew. Chem. Int. Ed.* **59**, 17607-17613 (2020).

15. Ding, R. et al. Highly efficient and selective gold recovery based on hypercross-linking and polyamine-functionalized porous organic polymers. *ACS Appl. Mater. Interfaces* **14**, 11803-11812 (2022).

16. Chen, Y. et al. Construction of porphyrin and viologen-linked cationic porous organic polymer for efficient and selective gold recovery. *J. Hazard. Mater.* **426**, 128073 (2022).

17. Guo, J. et al. Highly efficient and selective recovery of Au(III) from aqueous solution by bisthiourea immobilized UiO-66-NH_2: Performance and mechanisms. *Chem. Eng. J.* **425**, 130588 (2021).
